# PCW-A1001, AI-assisted *de novo* design approach to design a selective inhibitor for FLT-3(D835Y) in acute myeloid leukemia

**DOI:** 10.3389/fmolb.2022.1072028

**Published:** 2022-11-25

**Authors:** Seong Hun Jang, Dakshinamurthy Sivakumar, Sathish Kumar Mudedla, Jaehan Choi, Sungmin Lee, Minjun Jeon, Suneel Kumar Bvs, Jinha Hwang, Minsung Kang, Eun Gyeong Shin, Kyu Myung Lee, Kwan-Young Jung, Jae-Sung Kim, Sangwook Wu

**Affiliations:** ^1^ R&D Center, PharmCADD, Busan, South Korea; ^2^ Division of Radiation Biomedical Research, Korea Institute of Radiological and Medical Sciences, Seoul, South Korea; ^3^ Therapeutics & Biotechnology Division, Korea Research Institute of Chemical Technology, Daejeon, South Korea; ^4^ Department of Medicinal Chemistry and Pharmacology, University of Science & Technology, Daejeon, South Korea; ^5^ Department of Physics, Pukyong National University, Busan, South Korea

**Keywords:** FLT-3, AML, kinase inhibitors, de novo drug design, LSTM, AI

## Abstract

Treating acute myeloid leukemia (AML) by targeting FMS-like tyrosine kinase 3 (FLT-3) is considered an effective treatment strategy. By using AI-assisted hit optimization, we discovered a novel and highly selective compound with desired drug-like properties with which to target the FLT-3 (D835Y) mutant. In the current study, we applied an AI-assisted *de novo* design approach to identify a novel inhibitor of FLT-3 (D835Y). A recurrent neural network containing long short-term memory cells (LSTM) was implemented to generate potential candidates related to our in-house hit compound (PCW-1001). Approximately 10,416 hits were generated from 20 epochs, and the generated hits were further filtered using various toxicity and synthetic feasibility filters. Based on the docking and free energy ranking, the top compound was selected for synthesis and screening. Of these three compounds, PCW-A1001 proved to be highly selective for the FLT-3 (D835Y) mutant, with an IC_50_ of 764 nM, whereas the IC_50_ of FLT-3 WT was 2.54 μM.

## Introduction

Overexpression or mutation of some signaling proteins leads to cancer development (Kazi and Rönnstrand, 2019). Among the most mutated extracellular signaling mediators in cancer are the receptor tyrosine kinases (RTKs) (McDonell et al., 2015). Among the five known types of RTKs, FMS-like tyrosine kinase (Kindler et al., 2010) (FLT-3) belongs to type III. It plays an essential role in regulating early hematopoiesis because it is selectively expressed on CD34+ hematopoietic stem cells and immature hematopoietic progenitors (Rosnet et al., 1996; Kindler et al., 2010). It is also expressed in the liver, spleen, lymph nodes, thymus, placenta, gonads, and brain (Del Zotto et al., 2001; Stirewalt and Radich, 2003; Brown and Small, 2004). Our work is focused on FLT-3, a gene that is highly mutated in acute myeloid leukemia (AML) (Grafone et al., 2012).

Interestingly, thousands of mutations (mostly insertions) have been reported for FLT-3. Many FLT-3 point mutations are commonly found in AML, and the activation loop residue D835, which stabilizes the inactive conformation is the predominant site of mutations (Yamamoto et al., 2001; Liang et al., 2003; Smith et al., 2012). Overexpression and frequent FLT-3 mutations are associated with poor prognoses and AML pathogenicity and activate downstream signaling molecules, which leads to stimulation and survival of cancerous cells (Zhang and Broxmeyer, 1999; Hayakawa et al., 2000; Lin et al., 2012). Treating AML patients by targeting FLT-3 and its mutants with small molecules is considered a promising strategy (Assouline et al., 2012; Leung et al., 2013; Gebru and Wang, 2020; Ambinder and Levis, 2021).

Since approval by the Food and Drug Administration (FDA) of the first tyrosine kinase inhibitor imatinib (Savage and Antman, 2002) two decades ago, several drugs targeting FLT-3 have entered clinical trials. Nevertheless, only midostaurin and gilteritinib have been approved by the FDA (Scholl et al., 2020). FLT-3 inhibitors are classified as type I or type II based on their binding with the protein. Type I inhibitors such as sunitinib (Schittenhelm et al., 2006), midostaurin (Stone et al., 2004), lestaurtinib (Smith et al., 2004), crenolanib (Heinrich et al., 2012), and gilteritinib (Grunwald and Levis, 2013) bind with the active state (DFG-in) of FLT-3, whereas type II inhibitors such as sorafenib (Auclair et al., 2007), ponatinib (O’Hare et al., 2009), and quizartinib (Zarrinkar et al., 2009) bind only with the inactive (DFG-out) FLT-3 conformation (Scholl et al., 2020). Studies have shown that type I inhibitors are more promising for use in AML treatment, as they target the predominant mutated kinase (Wodicka et al., 2010; Smith et al., 2012). There has been tremendous interest in developing FLT-3 inhibitors using classic computer-aided drug design approaches (Chang Hsu et al., 2014; Ke et al., 2015). In this study, we focus on developing a more rational approach for preparation of FLT-3 type-I inhibitors.

Recent breakthroughs show the significance of artificial intelligence (AI) in drug discovery, and AI reduces costs and increases the speed of the drug discovery pipeline (Mak and Pichika, 2019). One of the main bottlenecks of traditional *de novo* drug design methods is the complicated synthetic routes; reported AI methods suggest synthetically feasible molecules or synthetic pathways that can help chemists (Corey and Wipke, 1969; Hessler and Baringhaus, 2018). Using AI, identification of a DDR1 kinase inhibitor was completed in just 60 days, including synthesis and experimental validation (Zhavoronkov et al., 2019). Excientia prepared the first AI-designed drug (DSP-1181) to treat obsessive-compulsive disorder (OCD), which subsequently entered clinical trials (Luo et al., 2022). They also discovered the AI-designed molecule EXS-21546 for immuno-oncology, which entered clinical trials in 8 months. Insilico medicine (www.insilico.com) used its AI program to develop a novel inhibitor (ISM001-055) for antifibrotic targets, and it reached clinical trials in 9 months. Recently, they have announced a preclinical candidate for the main protease of SARS-CoV-2, which was discovered with their novel AI platform, Chemistry42.

**FIGURE 1:**
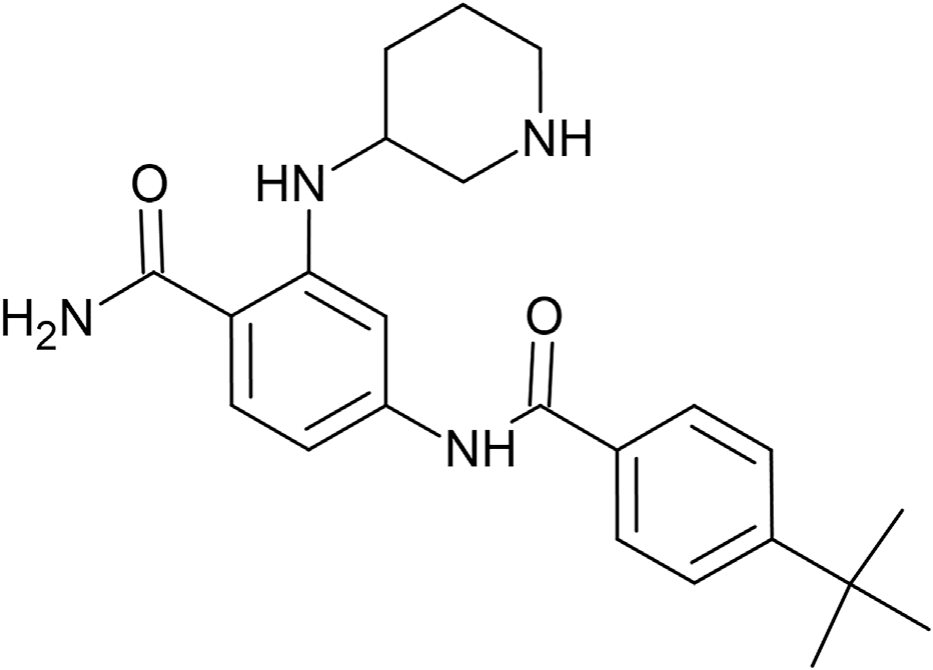
2D Chemical structure of FLT-3 inhibitor PCW-A1001.

Network-based approaches are widely used to infer relationships between diseases and drugs (Guney et al., 2016) and are more focused on predicting novel protein targets and new uses of known drugs (Berger and Iyengar, 2009; Wu et al., 2013). In the current study, we used our reverse network theory approach developed in-house to identify a potential therapeutic target for PCW-1001. Based on the network theory and docking results, FLT3 was considered a potential target. Further biological screening studies showed that PCW-1001 exhibited an inhibitory IC_50_ of 13.6 μM against FLT-3 WT and 1.83 μM against the FLT-3 (D835Y) mutant (Kang et al., 2022). An AI-assisted *de novo* design approach was applied to identify a potent and selective inhibitor for the FLT3/FLT-3 (D835Y) mutant. This parent compound (PCW-1001) was considered for further optimization, and more than 10,416 analogs were generated using the LSTM approach. These hits were further evaluated for synthetic feasibility by in-house machine learning models and assessed for potential structural alerts. The resulting hits were subjected to docking studies, binding mode reviews, and free energy calculations for prioritization. Based on the binding mode review and free energy calculation data, the top compound was prioritized for synthesis and further screening. Screening data showed that PCW-A1001 (Figure 1) proved to be a potential and selective inhibitor against the FTL3 (D835Y) mutant.

## Results and discussion

### Network-based reverse target prediction

**FIGURE 2:**
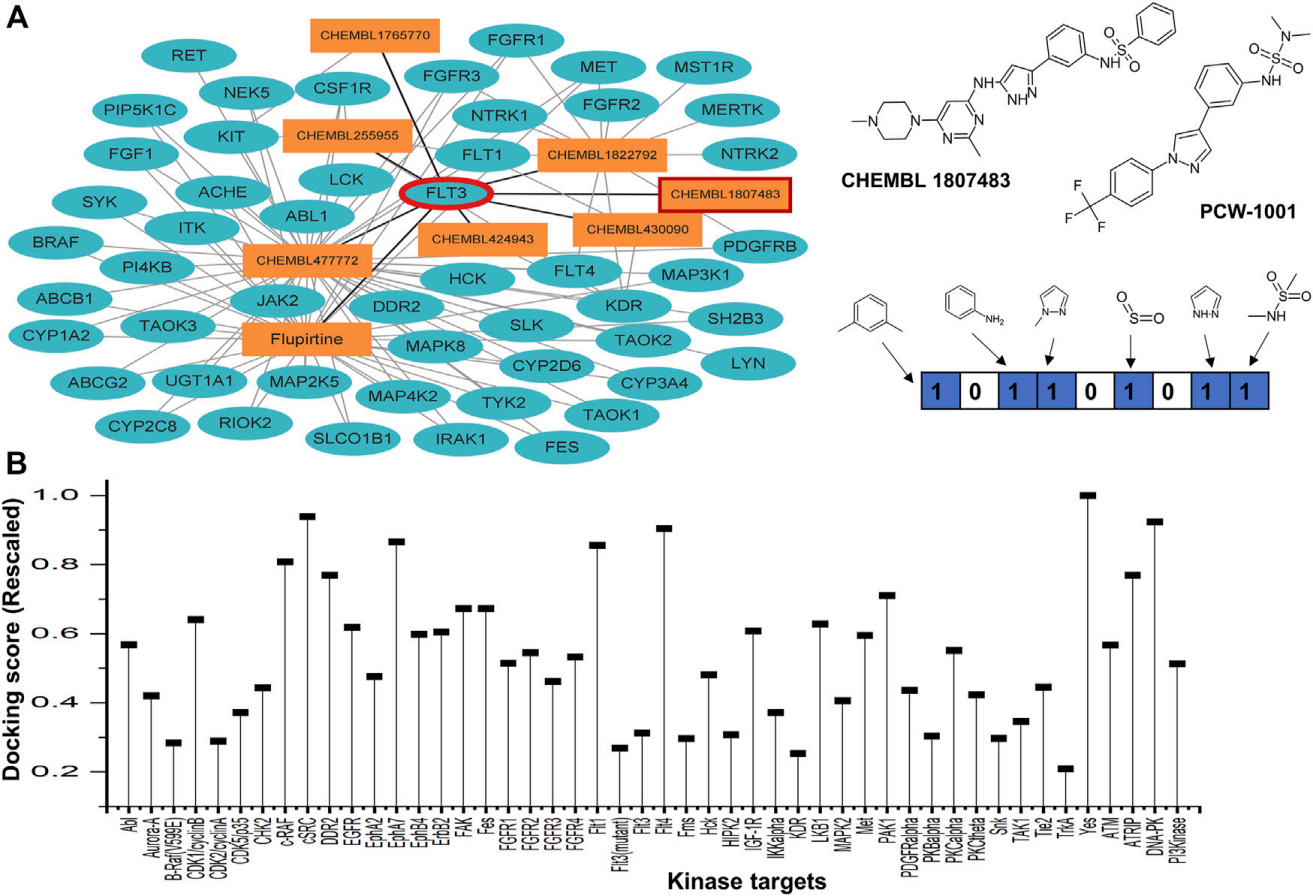
Network-based reverse target prediction. **(A)** Protein-Ligand interaction network with nodes (depicted in dark turquoise elliptical sphere) as proteins and edges (rectangular orange box) as ligands obtained from ChEMBL, DrugBank and PubChem. Tanimoto similarity identified using MACCS keys fingerprint between query compound (PCW-A1001) and the top hit CHEMBL 1807483 (shows interactions with FLT3) is 0.67. **(B)** Study of selectivity of the PCW-1001 against the panel of 48 representative kinase enzymes. The score was rescaled to be ranged, as the lower value corresponds to more energetically favorable one.

We implemented an in-house network-based reverse target prediction module to identify a protein target for PCW-1001 (Figure 2). Our parent compound, PCW-1001, exhibited a significant inhibitory profile against various breast cancer cell lines, but a substantial protein target was unknown (Kang et al., 2022). PCW-1001 compound structural similarity (atom pair descriptors) was computed against the ChEMBL chemical database. The generated similarity matrix of the ChEMBL database with PCW-1001 and its corresponding protein target information was considered for further analysis. Ensemble docking studies were carried out for PCW-1001 against all 2,000 unique targets [with a known crystal structure database (www.rcsb.org)]. Of the top 10 scored (docking score) targets, five kinases (FLT3, JAK2, NTRK, MKNK2, and TGFBR1) were observed to be potential targets for PCW-1001. All five kinase targets are known to play a critical role in treating various cancers; among the five targets, we selected FLT-3 based on the score. Furthermore, FLT-3 point mutations are frequently found in AML, where the mutations occur in the activation loop residue D835 and stabilize the active conformation.

### Artificial intelligence-assisted *de novo* design of a novel FMS-like tyrosine kinase 3 inhibitor using the long short-term memory approach

**FIGURE 3:**
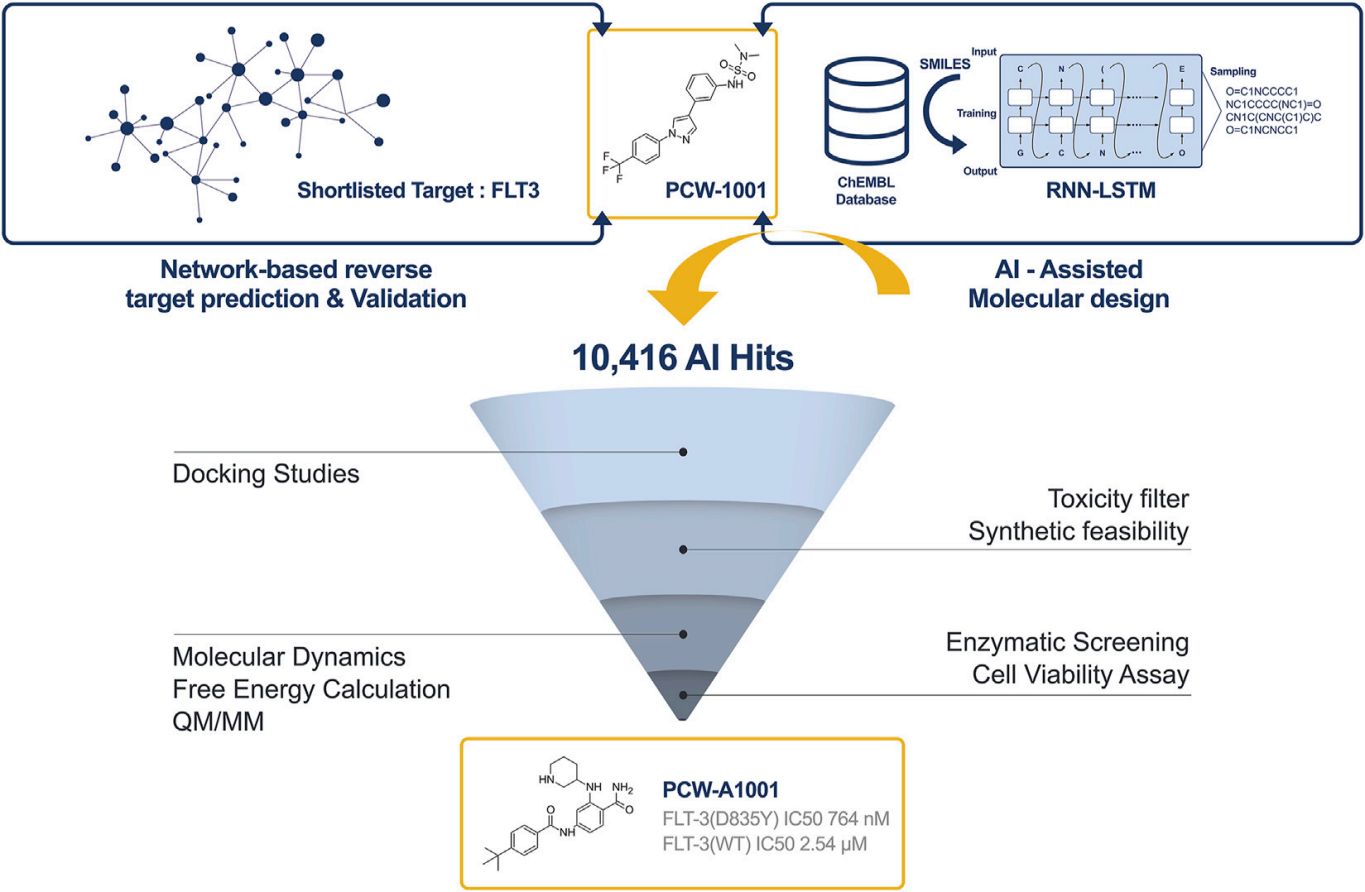
Overall pipeline of AI based drug discovery approach to identify PCW-A1001 from PCW-1001. Step-1: The target protein for the PCW-1001 was identified as FLT3 from the network analysis. Step-2: AI-assisted drug design using the RNN-LSTM method. Step-3: Generated compounds were evaluated using various lead-like identification filters. Step-4: Binding mode analysis (Docking, Molecular dynamics, QM/MM, Free energy calculation) of the filtered molecules. Step-5: Synthesis and characterization. Step-6: *In vitro* Assay for the enzymatic activity and cell viability.

In the current study, we applied a deep recurrent neural network (RNN) with long short-term memory (LSTM) cells for *de novo* drug design (Merk et al., 2018) to generate potential hit candidates around PCW-1001 (Figure 3). We fine-tuned the model by using the transfer learning approach to optimize *de novo* generation of FLT-3 active compounds (Kang et al., 2022). We sampled 10,416 SMILES (Simplified Molecular Input Line Entry System) strings from 20 epochs from the resulting fine-tuned model. AI-generated hits were further evaluated using MOSES (Polykovskiy et al., 2020) for novelty, validity, diversity, scaffold similarity, and uniqueness. Benchmarking analyses indicated that 98.8% of the hits were valid, 85% of the hits were unique, and 90% of the hits were novel. The chemical space of AI-generated hits falls within the range of FLT3 known actives and PCW-1001 (Supplementary Figure S1). Furthermore, violin plot analysis also suggested that the distribution of molecular weights and LogP of AI-generated hits were within the range of known FLT3 actives (Supplementary Figure S2). Overall, AI-generated hits fell within the chemical space of known actives, and MOSES analysis suggested that AI-generated hits were diverse and novel compared with known FLT3 actives and PCW-1001.

Based on the binding analysis and interaction pattern studies, we identified 1750 compounds out of 10,416 hits as suitable for further studies. Following this preliminary evaluation, we assessed the resulting structures for toxicity endpoints *via* our Pharmulator™. We generated 9 toxicity models (different end-points) using available literature data, and validated models were deployed in Pharmulator™ to assess the hit moieties quickly. Of 1750 hits, only 190 compounds passed synthetic feasibility, novelty, drug-like, all toxicity, and PAINS filters and were further subjected to a binding pose analysis and free energy calculations. We selected the top compound for synthesis and *in vitro* screening.

### Synthesis and structural characterization of the *de novo* compound PCW-A1001

**SCHEME 1:**
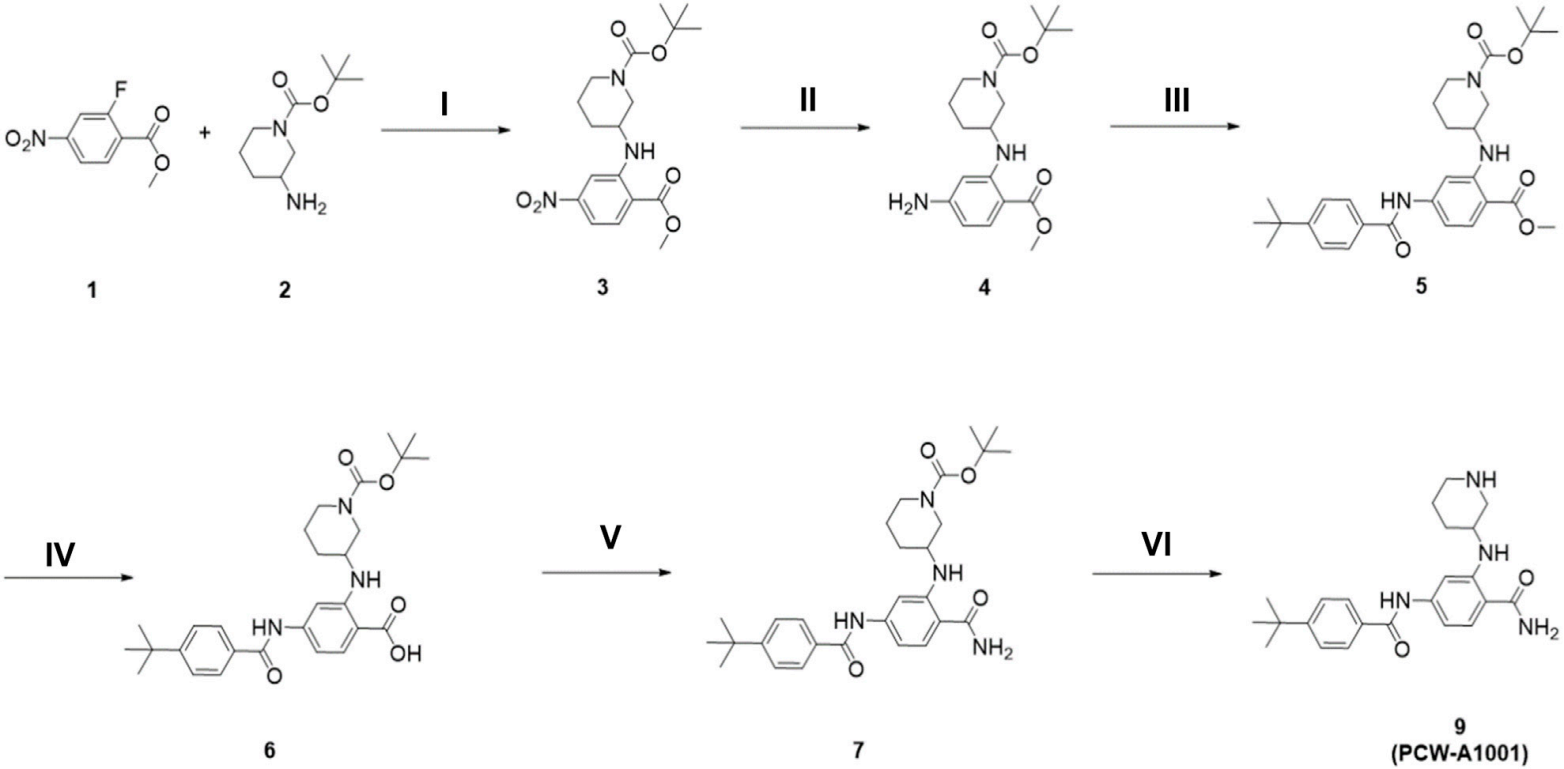
The synthesis of PCW-A1001. (i) K_2_CO_3_, DMF, 70°C, 12 h; (ii) H_2_(g), Pd/C, EtOAc, r.t., 10 min; (iii) 4-tert-butylbenzoic acid, EDCI, DMAP, DCM, r.t., 48 h; (iv) LiOH·H_2_O, THF/MeOH/H_2_O, r.t., 12 h; (v) NH_4_Cl, HBTU, DIPEA, DMF, r.t., 12 h; (vi) 20% TFA in MeOH, r.t., 3 h.

The synthetic route to PCW-A1001 is summarized in Scheme 1. Methyl 2-fluoro-4-nitrobenzoate **1** and tert-butyl 3-aminopiperidine-1-carboxylate **2** were reacted in the presence of K_2_CO_3_ to obtain **3** through nucleophilic aromatic substitution. The nitro group of resulting compound **3** was converted to an amino functional group *via* hydrogenation. Amine Compound **4** was coupled with 4-(*tert*-butyl)benzoic acid in the presence of EDCI and a catalytic amount of DMAP and then hydrolyzed using LiOH·H_2_O to produce intermediate **6**. The acid functional group was efficiently converted to an amide with the HBTU coupling reagent. The Boc protecting group of the secondary amine in the piperidine ring was removed to obtain the desired compound PCW-A1001. The step-by-step synthesis and structural characterizations are shown in the Supplementary Material.

### Structural interaction and stability analysis of PCW-A1001 with wild-type FLT-3 and the FLT-3 (D835Y) mutant

**FIGURE 4:**
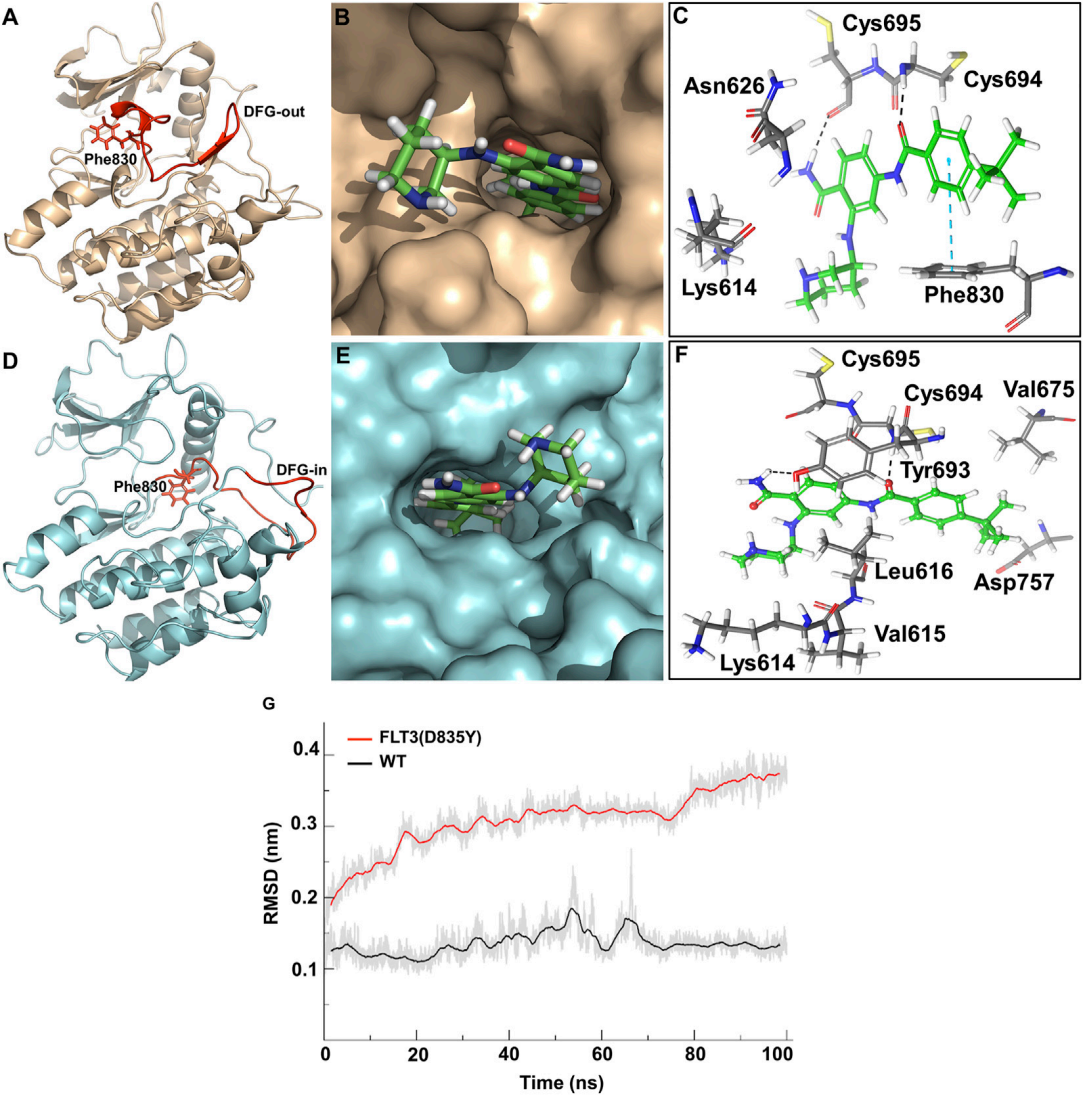
Binding analysis of PCW-A1001. **(A)** DFG-out conformation of FLT-3 wild type **(B)** Binding mode of PCW-A1001 with FLT-3 wild type **(C)** Crucial interactions observed in the FLT-3 wild-type (sticks). **(D)** DFG-in conformation of FLT-3 (D835Y) mutant, **(E)** Binding mode of PCW-A1001 with FLT-3 (D835Y), **(F)** Crucial interactions observed in the FLT-3 (D835Y) (sticks). Hydrogen bond interactions are represented in black dashed lines and pi-pi interactions in the blue dashed lines. **(G)** RMSD (Cα) plot of FLT-3 wild type and D835Y mutant.

The top predicted binding mode of PCW-A1001 with FLT-3 WT showed two key hydrogen bonding interactions with Cys694 and Cys695 and a π-π interaction with Phe830. The docking complex of PCW-A1001 with FLT-3 WT was considered for molecular dynamics (MD) simulation for 100 ns. The MD simulation results also showed that the compound binding interactions observed in the initial docked complex were retained in PCW-A1001. The compound bound perfectly in the ATP binding site by forming hydrogen bonds with the two cysteine residues (Cys694 and Cys695) located in the hinge region. As observed in several inhibitor-kinase complexes, hydrogen bonding interactions with the inhibitor are essential for kinase inhibitory activity (Banks et al., 1979; Ke et al., 2015). The carbonyl moiety of the benzamide group formed a hydrogen bond with the NH group of the Cys694 residue (Figure 4).

The NH moiety of the next benzamide group in the compound formed a hydrogen bond with the backbone carbonyl group of the Cys695 residue. Phe830 in the DFG loop in wild-type (WT) FLT-3 formed π-π interactions with compound PCW-A1001. The binding free energy of PCW-A1001 was −13.4 kcal/mol with FLT-3 WT but −14.8 kcal/mol with the FLT-3 (D835Y) mutant, whereas those of the precursor compound PCW-1001 were −7.2 kcal/mol and −8.07 kcal/mol for FLT-3(WT) and FLT-3 (D835Y), respectively (Table 1).

**TABLE 1:** Binding free energy calculation for PCW-A1001 with WT and mutant FLT-3.

Compound	FLT-3 (WT)	FLT-3 (D835Y) mutant
PCW-A1001	−13.4 kcal/mol	−14.8 kcal/mol
PCW-1001	−7.2 kcal/mol	−8.07 kcal/mol

In the FLT-3 (D835Y) mutant, the NH group of Cys694 formed a hydrogen bond with the CO moiety of the benzamide group in PCW-A1001, as seen with WT FLT-3. Cys695 also maintained its hydrogen bonding interactions, as in the WT; additionally, Lys614 and Tyr693 interacted with the protein. The Cα-RMSD of the WT FLT-3 and the mutant complex showed that the complex was stable throughout the simulation (Figure 4).

Based on the binding study of PCW-A1001 and its precursor compound PCW-1001 against the panel of kinase enzymes, selectivity was achieved by PCW-A1001 for FLT-3 (D835Y). The selectivity scores of PCW-A1001 and PCW-1001 were calculated from the dock score (rescaled) of the selected kinase panel of enzymes as 0.33 and 0.46, respectively, for the Flt-3 (D835Y) mutant (Supplementary Figure S3).

### QM/MM analysis of PCW-A1001

**FIGURE 5:**
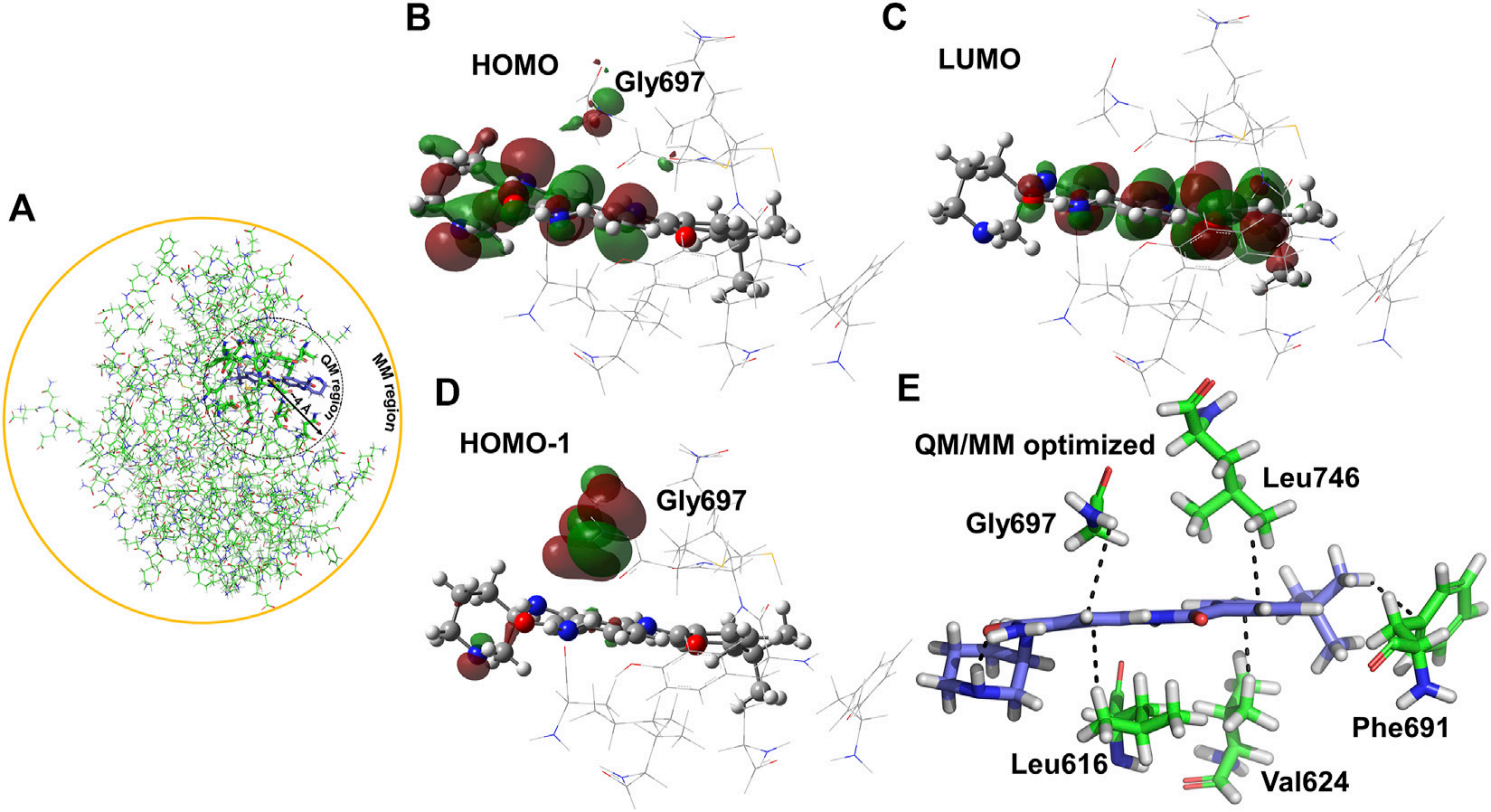
QM optimization of PCW-A1001 with FLT-3(D835Y). **(A)** Schematic representation of the QM and MM optimization region selected in the protein-ligand complex of the FLT-3-D835Y mutant. **(B,D)** HOMO/HOMO-1 surface of FLT-3 (D835Y) and **(C)** LUMO surface in FLT-3 (D835Y) in complex with PCW-A1001. Ligand PCW-A1001 shows in ball and sticks and the residues in the QM region shown in lines. **(E)** Hydrophobic interactions observed in the QM/MM optimized complex.

QM/MM optimization was used to validate the interactions between PCW-A1001 and the FLT-3 (D835Y) mutant in the MD-determined complex to study the electronic and structural properties of the ligand and selected atoms of the protein (Figure 5). The electrostatic and van der Waals interactions were intact in the protein–ligand complex, as with the MD structure. The ligand was stabilized at the binding site through hydrogen bonding and -CH-π and -NH-π interactions. The backbone -NH group from Gly697 interacted with the phenyl ring of the ligand. In addition to hydrogen bonding interactions, -CH-π interactions were dominant in the complex formed between the ligand and protein. The Leu746, Phe691, Val624, and Leu616 residues were involved in the -CH-π interaction, as shown in Figure 5. The same pattern was also observed in the case of WT protein binding with the ligand, except for the -CH-π interactions with Ala642. The calculated interaction energies for the ligand and protein were −46.91 and −34.04 kcal/mol for the mutant and WT, respectively. These binding affinities were in good agreement with the free energy calculations for an explicit water environment.

**FIGURE 6:**
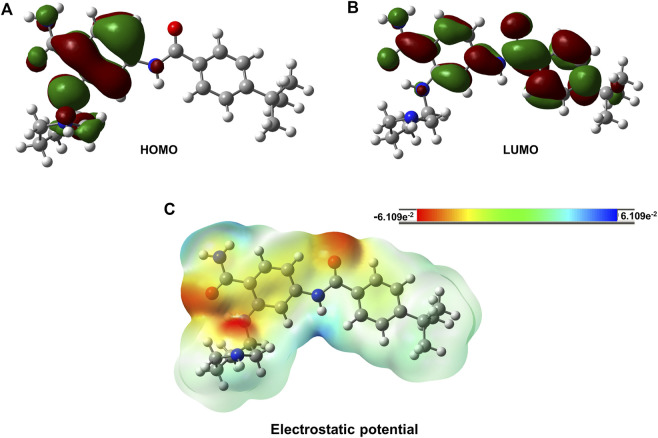
Isosurfaces of Frontier orbitals HOMO (Highest Occupied Molecular Orbital) and LUMO (Lowest Unoccupied Molecular Orbital). The charge distribution over PCW-A1001 molecule. **(A)** Isosurface of HOMO, **(B)** Isosurface of LUMO and **(C)** Electrostatic potential of the PCW-A1001. The red color indicates the negative charge and green color indicates the positive charge for the PCW-A1001. The electrostatic potential values were distributed from −6.109e^−2^ to 6.109e^−2^.

The electron distribution was determined from the calculated orbital energies. The highest occupied molecular orbital (HOMO), HOMO-1, and lowest unoccupied molecular orbital (LUMO) were computed and are displayed in Figure 6. The HOMO surface was predominantly localized in the hydrogen bonding interaction regions of the ligand, whereas the LUMO surface was distributed evenly across the ligand (Figure 6). Gly697, which was involved in the -NH-π interaction with the ligand, contributed less to the HOMO, whereas HOMO-1 was highly localized on Gly697. The piperidine ring in the ligand formed an intramolecular hydrogen bond and stabilized the ligand orbitals. Thus, the HOMO was localized on and near the piperidine ring. Furthermore, atomic charges were calculated with natural population analysis. The sum of the atomic charges on the ligand was found to be 0.02 au. No significant charge transfer from the ligand to the protein was observed.

We also analyzed the Frontier orbitals of PCW-A1001, as shown in Figure 6. Frontier orbitals direct the mode of interaction between drugs and proteins. The HOMO and LUMO contribute to the chemical stability of the molecule. If the energy gap is zero or negligible, the molecule is highly reactive. PCW-A1001 was stable and showed an energy gap of 3.7 eV. The HOMO was localized on the phenyl ring, and the LUMO was distributed across two phenyl rings of PCW-A1001. This indicated that intramolecular charge transfer might enhance the stability of PCW-A1001. The molecular electrostatic potential illustrates the charge distribution of a molecule. This explains how one molecule can interact with another. The electrostatic potential helps determine the electrophilic and nucleophilic sites involved in hydrogen bond formation. The calculated electrostatic potential surface is shown in Figure 6. The positive and negative potentials are indicated by blue and red colors, respectively. Atoms in the positive potential region act as electron acceptors, whereas atoms with a negative potential behave as electron donors during hydrogen bond formation with FLT-3. The aromatic phenyl rings involved in -CH-π interactions were found between the positive and negative potentials. The results show that the charge distribution over PCW-A1001 was favorable for interacting with the binding pocket of FLT-3.

### Inhibition of MV4-11 and acute myeloid leukemia cell lines by PCW-A1001

**FIGURE 7:**
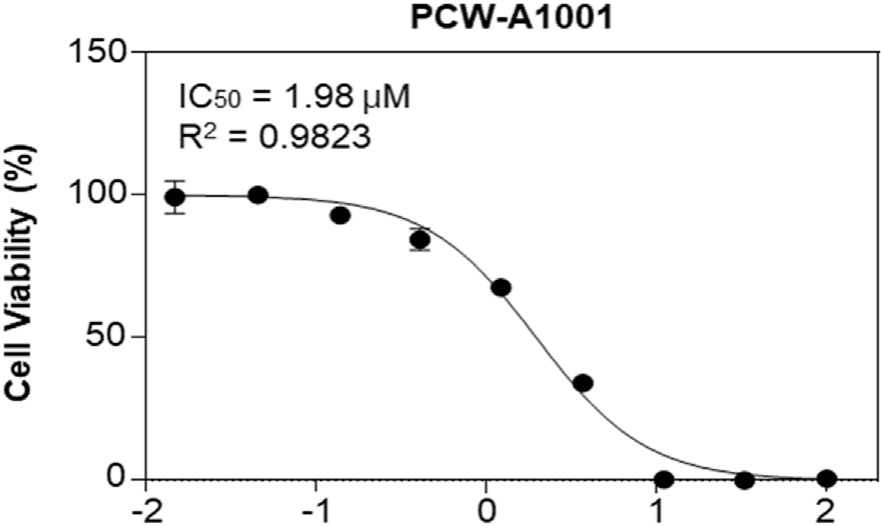
PCW-A1001 inhibits the viability of AML cell line. MV4-11 cells were treated with PCW-A1001 for 72 h, and cell viability was analyzed. The data are presented as the mean ± standard error mean. The dose response curve was generated using OriginPro 2021 (OriginLab, United States).

MV4-11 cells and FLT-3-mutated AML cells (Quentmeier et al., 2003) were used to examine the anticancer activity of PCW-A1001. It inhibited the proliferation of MV4-11 cells, with an IC_50_ of 1.98 μM, showing that PCW-A1001 has potent anticancer activity in AML cells (Figure 7).

### *In vitro* kinase activity of PCW-A1001

**FIGURE 8:**
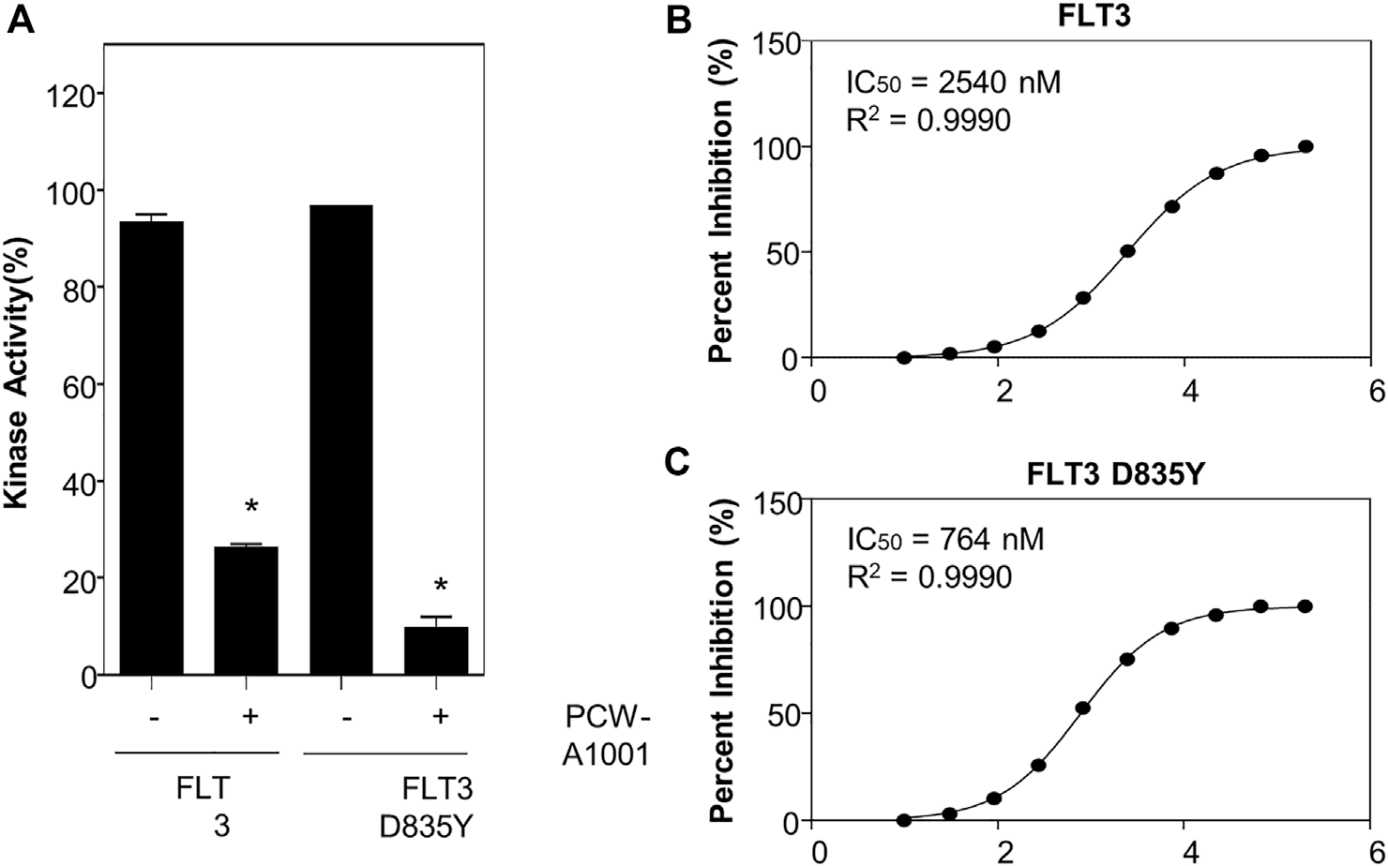
PCW-A1001 inhibits the kinase activity of FLT-3 WT and D835Y mutant. **(A)** PCW-A1001 inhibited FLT-3 WT and D835Y mutant kinase at 10 µM **(A)**. IC50 of PCW-A1001 for FLT-3 WT **(B)** and D835Y mutation **(C)** were analyzed by ThermoFisher. The data are presented as the mean ± standard error mean. *p < 0.01.

Next, we performed an *in vitro* kinase assay to evaluate the inhibitory activity of PCW-A1001 for WT FLT-3 and the D835Y-mutant kinase. Interestingly, our data indicated that PCW-A1001 inhibited the mutant kinase more effectively than the WT (Figure 8A). The IC_50_ determined from the kinase assay was 764 nM for the FLT-3 D835Y mutant, which was only one-third the IC_50_ for WT (2.54 μM) (Figures 8B,C). Thus, our data provide proof-of-concept evidence for the AI-assisted *de novo* drug design approach.

## Materials and methods

### Reverse target prediction—a network-based approach

The input structures were used as SMILES structures and converted into fingerprints. We used six kinds of fingerprints: RDKit, MACCSkeys, AtomPair, Torsion, Morgan, and Morgan with Features (Landrum, 2016). A total of six fingerprints and six similarities were calculated and compared with the precalculated scores for the ligands in our protein–ligand interaction network database. A total of 10,647 compounds were used for the analysis among 262,327 compounds in the ChEMBL database. The list of compounds used with SMILES is included in the (Supplementary Table S2). In detail, the similarities between the input compound and all drugs in the network were calculated. Here, we used six similarity measures: Tanimoto, Dice, Sokal, Cosine, Kulczynsk, and McConnaughey. We selected drugs with high similarity scores (sum of similarity values). Using protein–drug relationships in the network, the similarity score of a drug was assigned to all proteins connected to the drug. Then, we obtained a list of proteins with similarity scores for each fingerprint. Based on the similarity score, the list was sorted in descending order, and a rank value, which is a descending value from the maximum rank, was assigned. The rank score was calculated using the following formula:

$$rank\;score=\frac{rank\;value}{\sum rank\;values}$$ (1)

The proteins obtained a total rank score R, which was the sum of the rank score of each fingerprint. The rank score was further modified as $\tilde{R}$ by applying the following formula and assigned to each protein. The modified total rank score showed the potential of the target protein. The network model was validated using eight known Bruton’s tyrosine kinase (BTK) inhibitors, the model prediction rank was given (Supplementary Figure S4), and the details of the targets along with their inhibitors are given (Supplementary Table S1).

$$\tilde{R}=\frac{1}{R_{\max}}\sum_{Fingerprint}rank\;score$$ (2)

### AI-assisted *de novo* design

A recurrent neural network (RNN) is a type of neural network that is widely used for natural language processing (NLP) tasks from simple language processing to complex cheminformatics problems. RNNs have been successfully applied for protein structure and function predictions from sequences (Liu, 2017; Zhang et al., 2018), property predictions, fragment-based hit generation (Awale et al., 2019), and hit identification (Segler et al., 2018; Erikawa et al., 2021).

For *de novo* drug design, we successfully applied generative recurrent neural networks (RNN) containing long short-term memory (LSTM) cells (Merk et al., 2018). The model considers the SMILES strings for molecular representation and learns the patterns and their probabilities from pretraining for use in generation of the SMILES structures. We fine-tuned the generated structures (SMILES) for specific molecular targets or chemical series by employing transfer learning. The generative LSTM approach has proven helpful in low-data drug discovery, hit expansion, molecular design (fragment-based), and lead optimization (Gupta et al., 2018; Segler et al., 2018; Erikawa et al., 2021).

All deep learning models were applied using TensorFlow (v2.1, www.TensorFlow.org) in Python (v3.7, www.python.org). We have used RDkit (www.rdkit.org) for most cheminformatics activities (property calculations, SMILES string validity calculations, molecular fingerprint calculations, and molecular clustering calculations). A detailed analysis of the generated SMILES strings was performed using the Jupyter notebook (www.anaconda.org).

RNNs were used as autoencoders, and the deep learning model employed for this study was initially pretrained to capture the structures of 438,552 bioactive small molecules (after carefully excluding the FLT-3 actives) retrieved from ChEMBL25 (KD, Ki, EC50, IC50 < 1 μM) and represent them as simplified molecular-input line-entry system (SMILES) strings (Weininger, 1988). Using this pretrained model, we fine-tuned the model by transfer learning to bias *de novo* molecule generation toward the desired bioactivities of the templates (Merk et al., 2018). This fine-tuning step was employed to train the model for designing functional mimetics. Generated hits were further evaluated and benchmarked using Molecular sets (MOSES) (Polykovskiy et al., 2020).

### Synthetic feasibility

The synthetic feasibility of each compound generated by AI was obtained with the retrosynthesis-associated fragment-based synthetic feasibility (RAFSF) score module. The fundamental idea of the module was that after cleaving synthetically meaningful bonds of the given compound, the bonds and the resulting fragments were searched from a bond/fragment space extracted from the ChEMBL (Mendez et al., 2019) or USPTO (Lowe, 2017) grants database in the same way. In the ChEMBL small molecule database, 1,917,863 molecules with molecular weights of less than 1,000 were used, as were 1,808,937 reactions from the USPTO grants database. To break bonds, we used the modified BRICS (Degen et al., 2008) module included in the rdkit (Landrum, 2016). If the bond/fragment from the given compound was not contained or rarely appeared in the bond/fragment space, a RAFSF score with a high value was assigned, meaning it was synthetically unfeasible. The RAFSF score is a value ranging from 1 (highly feasible synthesis) to 10 (highly unfeasible).

### Protein preparation and modeling

The DFG-out WT FLT-3 protein structure was downloaded from the protein databank (www.rcsb.org) with PDB ID: 1RJB in the DFG-out conformation. Protein structures were prepared by correcting the bond orders, adding missing hydrogens, optimizing H-bonding with the protonation states of residues at pH 7.0, and restraining minimization for added hydrogens using the OPLS2005 forcefield of Protein Preparation Wizard (Sastry et al., 2013). The DFG-in conformation of the FLT-3 (D835Y) mutant was modeled using Modeler 9.25 (Sali and Blundell, 1993) with two templates, as reported previously (Ke et al., 2015)**.** The first template was the DFG-out conformation of FLT-3 (PDB: 1RJB) with its DFG motif removed, and the other template was the DFG-in conformation of the colony-stimulating factor-1 receptor (CSF-1) crystal structure (PDB id: 3LCD). These templates shared 93% and 63% sequence identity with the target protein, respectively. The model was subjected to loop refinement and minimization, followed by validation using a standard protocol discussed elsewhere (Sivakumar et al., 2013; Ke et al., 2015).

### Ligand preparation and molecular docking

Hit compounds were initially optimized using the DFT method in Gaussian 16 with B3LYP functionals and the 6-31G** basis set (Tirado-Rives and Jorgensen, 2008). The antechamber obtained GAFF atom types with RESP charges from the Gaussian output file. The atom types and all needed parameters for the ligand were obtained from the above process along with parmed and tleap (Shirts et al., 2017). Molecular docking was carried out using Glide XP (Friesner et al., 2006) with default parameters; initially, the receptor was prepared with a grid box set covering the centroid of the active site, followed by flexible ligand sampling of the ligand docking.

### Molecular dynamics simulations

The stabilities of the complexes were studied by MD simulations using Gromacs 2019 (Abraham et al., 2015). The Charmm36 force field (Huang and MacKerell, 2013) was used for the protein parameters. The protein–ligand complexes were solvated explicitly using the TIP3P water model inside the cubic box, and their sizes extended 0.1 nm away from the protein on the edges of the box in each direction. The overall charge of the system was neutralized by adding a 0.15 M salt (Na^+^Cl^−^). All simulations were carried out on GPU-enabled Linux clusters. The entire system was minimized with a maximum step size of 50,000 until the maximum force was less than 10 kJ/mol. The system was then equilibrated for 5 ns under NVT conditions with temperature coupling for two separate groups, protein–ligand and water-ions, at 300 K. The Lincs algorithm was used to constrain the bonds of the hydrogen atoms (Hess et al., 1997). A Berendsen thermostat and V-rescale were used to keep the temperature and pressure constant, respectively (Lemak and Balabaev, 1994; Bussi et al., 2007). The cutoff distances for Coulomb and van der Waals interactions were set as 1.2 nm. The particle mesh Ewald method (PME) was used to calculate the long-range electrostatic interactions (Darden et al., 1993). The final production run was carried out for 100 ns at a temperature of 300 K and a pressure of 1 bar.

### Free energy calculation

The binding free energies for protein and ligand complexes were calculated in an explicit water environment by employing the alchemical method (Supplementary Figure S5). The final snapshot from the MD simulations (100 ns) was used as the starting point for the free energy simulations. The alchemical method involves two steps: 1) decoupling of the ligand from the protein–ligand complex in an explicit water environment and 2) decoupling of the ligand from the water environment. The decoupling process includes turning off the van der Waals and electrostatic interactions responsible for complex formation (protein–ligand or water-ligand) with the help of the coupling parameter (*λ*). First, electrostatic interactions were turned off slowly, while the van der Waals interactions were still present. Then, the van der Waals interactions between the protein and ligand (water and ligand) were turned off using the coupling parameter (*λ*). The electrostatic interactions were turned off by changing *λ* (0 0.25 0.5 0.75 1.0) from 0 to 1 with a step size of ∆λ = 0.25, and the van der Waals interactions were turned off with nonuniformly distributed values of *λ* (0.05, 0.1, 0.2, 0.3, 0.4, 0.5, 0.6, 0.65, 0.7, 0.75, 0.8, 0.85, 0.9, 0.95, 1.0). The same procedure was applied to decouple the ligands from the protein–ligand complex and ligands from the water environment. Therefore, 21 windows, each of 1 ns, were employed to decouple the ligand from the protein–ligand and water-ligand complexes. The free energy difference between the two end states was calculated using the Bennett acceptance ratio (BAR) method (Bennett, 1976). The BAR method is used to estimate the free energy difference between two states with the following equation:

$$\langle \frac{1}{1+\exp\left\{\beta \left({∆U}_{ij}-∆G\right)\right\}}\rangle i=\langle \frac{1}{1+\exp\left\{\beta \left(-{∆U}_{ij}+∆G\right)\right\}}\rangle j$$ (3)

where *β* is the reciprocal of the thermodynamic temperature, ΔG is the free energy difference between states i and j, and ΔU_*ij*_ = U_*j*_−U_*i*_ is the potential energy difference.

At each λ-point, the structures were subjected to energy minimization using the steepest descent method. Using Langevin dynamics, the resulting structures were equilibrated in an isothermal-isobaric (NPT) ensemble at 300 K and at a pressure of 1 bar. The rest of the simulation protocol was similar to the protocol followed in the classic MD section.

### QM/MM approach

The final snapshots of protein and ligand complexes determined from MD simulations were optimized in the gas phase at the (B3LYP-D3/6-31G*)/Universal force field level of theory with the help of the Gaussian16 package. It has been found in earlier studies that density functionals such as M06-2X, B3LYP-D, and ωB97XD are suitable for investigating noncovalent interactions. Hence, in all calculations, the QM region was optimized with dispersion-corrected B3LYP with the Grimme empirical dispersion functional (B3LYP-D3) using the 6-31G* basis set. The ligand and surrounding region within 4 Å were treated as the QM region, and the remaining parts were considered the MM region. We extracted only the QM region from the optimized geometries and added terminal hydrogens to calculate binding affinities. The resulting structures were used to calculate the interaction energies with the supermolecule approach at the B3LYP-D3/6-31G* level of theory.

IE=EC−(EM1+EM2) (4)

where IE is the interaction energy of the complex, EC is the energy of the complex, EM1 is the energy of the protein part of the complex, and EM2 is the energy of the ligand in the complex. All IEs were corrected for basis set superposition error (BSSE) using the counterpoise method suggested by Boys and Bernadi (Gutowski et al., 1993), as implemented in the Gaussian16 package (Frisch et al., 2016).

### Selectivity

In total, 49 kinases were evaluated *via* selectivity score calculation, and those 49 kinases were previously used for the actual kinase panel assay. Representative PDB structures for the 49 kinases were extracted from the RCSB Protein Data Bank (https://www.rcsb.org/). The ligands were docked to the binding pocket of each PDB using AutoDock-Vina (Trott and Olson, 2010). The resulting docking score was rescaled to observe and compare compound trends. Quantitatively, the selectivity score was calculated to measure the overall selectivity across different kinase families. A lower selectivity score indicates better selectivity for the tested compound.

$$Selectivity\;score\;\left(S\right)=\frac{number\;of\;kinases\;with\;rescaled\;docking\;score\;less\;than\;0.5}{total\;number\;of\;kinases\;tested}$$ (5)

### Cell culture and cell viability assay

MV4-11 cells were purchased from American Type Culture Collection (ATCC, VA, United States). The cells were passaged for less than 1 month, and mycoplasma infection was checked by PCR once a week. The growth medium was Iscove’s Modified Dulbecco’s Medium (IMDM; ThermoFisher, United States) supplemented with 10% fetal bovine serum (FBS; Corning, United States) and 1% penicillin/streptomycin (GenDEPOT, United States). The cells were maintained in a humidified atmosphere with 5% CO_2_ at 37°C. Cell viability was determined using the WST-8 assay (Cyto XTM cell viability assay kit; LPS solution, Daejeon, South Korea) in accordance with the manufacturer’s protocol.

### Synthesis of PCW-A1001

Unless otherwise stated, all reactions were performed under an inert (N_2_) atmosphere. Reagents and solvents were reagent grade and purchased from Sigma-Aldrich, Alfa Aesar, and Combi-Blocks. Anhydrous solvents were purchased from Sigma-Aldrich and used as provided. Reactions were monitored by TLC and visualized with a UV lamp and/or KMnO_4_ staining. Silica gel 60 (230–400 mesh, Merck) was used for flash column chromatography. ^1^H and ^13^C NMR spectra were recorded on BRUKER Ultrashield 300 and 400 MHz NMR spectrometers at 25°C. Chemical shifts are reported in parts per million (ppm). Data for ^1^H NMR are reported as follows: chemical shift (δ ppm) [multiplicity, coupling constant (Hz), integration]. Multiplicities are reported as follows: s = singlet, d = doublet, t = triplet, q = quartet, dd = doublet of doublets, m = multiplet. Data from ^13^C spectra are reported as chemical shifts (δ ppm). The residual solvent peak was used as an internal reference. Mass spectra were obtained on Acquity™ Waters A06UPD9BM and Agilent Technologies SG12109048 systems. Prior to biological testing, the final compound was confirmed to be > 98% pure by UPLC chromatography using a Waters ACQUITY H-class system fitted with a C_18_ reverse-phase column (ACQUITY UPLC BEH C_18_: 2.1 mm × 50 mm, Part No. 186002350) according to the following eluent conditions: (A) H_2_O + 0.1% formic acid, (B) CH_3_CN + 0.1% formic acid, (C) MeOH + 0.1% formic acid; (Ι) a gradient of 95% A to 95% B over 5 min; and (Ⅱ) a gradient of 95% A to 95% C over 5 min.

## Conclusion

In this work, we used an AI-assisted *de novo* drug design (LSTM) approach to identify a novel FLT-3 inhibitor that selectively targets the FLT-3 (D835Y) mutant. The deep learning model was pretrained on a known bioactive chemical space (ChEMBL22), and the generated hits were fine-tuned using our in-house FLT-3 inhibitors. The generated hits were further evaluated and filtered using various parameters focusing on their novelty, similarities, diversities, etc. We further evaluated the toxicities of the *de novo* molecules with our in-house program Pharmulator™. Among the screened hits, only 146 compounds passed the toxicity filters. The binding affinities, conformations and interaction patterns of these screened compounds were studied with WT FLT-3 and its mutant (D835Y). Since the FLT-3 (D835Y) mutant structure in the DFG-in conformation was unavailable, we modeled the protein to validate the compounds in terms of the binding interactions. The stabilities of complexes were further validated qualitatively with MD simulations and quantitatively with free energy calculations.

The top compound, named PCW-A1001, was considered for synthesis and screening studies. The anticancer activity was tested against MV4-11 cells to verify the effectiveness of these compounds in AML treatment. PCW-A1001 was found to be a promising inhibitor of FLT3, and it showed an IC_50_ of 764 nM against the FLT-3 (D835Y) mutant and 2.54 μM against WT FLT-3. PCW-A1001 also showed an IC_50_ of 1.98 μM against MV4-11-cell line screening. We successfully implemented reverse network theory and AI-based *de novo* design strategies and identified a potential inhibitor of the FLT3/FLT3 (D835Y) mutant, PCW-A1001. AI generated a hit, PCW-A1001 exhibited better activity than the parent compound, PCW-1001. Further fine-tuning of PCW-A1001 is in progress to optimize the selectivity and activity and will be reported in due course.

## Data Availability

The original contributions presented in the study are included in the article/Supplementary Material, further inquiries can be directed to the corresponding authors.
