# Supplementary material for: PCW-A1001, AI-assisted de novo design approach to design a selective inhibitor for FLT-3(D835Y) in acute myeloid leukemia
Source: Front Mol Biosci. 2022 Nov 25;9:1072028. doi: 10.3389/fmolb.2022.1072028 (PMC9732455; doi:10.3389/fmolb.2022.1072028)
Supplement: Supplementary file 2 [file DataSheet1.docx]

Supplementary file

**PCW-A1001, AI-assisted de novo design approach to design a selective inhibitor for FLT-3(D835Y) in acute myeloid leukemia**

Seong Hun Jang^1^, Dakshinamurthy Sivakumar^1^, Sathish Kumar Mudedla^1^, Jaehan Choi^1^, Sungmin Lee^1^, Minjun Jeon^1^, Suneel Kumar BVS^1^, Jinha Hwang^1^, Minsung Kang^2^, Eun Gyeong Shin^3,4^, Kyu Myung Lee^3^, Kwan-Young Jeong^3,4*^, Jae-Sung Kim^2*^, Sangwook Wu^1,5*^

^1^R&D Center, PharmCADD, 331, Jungang-daero, Dong-gu, Busan, 48792, Korea. ^2^Division of Radiation Cancer Research, Korea Institute of Radiological and Medical Sciences, Seoul, Korea. ^3^Therapeutics & Biotechnology Division, Korea Research Institute of Chemical Technology, Daejeon, 34114, Korea. ^4^Department of Medicinal Chemistry and Pharmacology, University of Science & Technology, Daejeon, 34113, Korea. ^5^Department of Physics, Pukyong National University, Busan, 48513, Korea.

*Corresponding authors: Kwan-Young Jung: krjeong@krict.re.kr, Jae-Sung Kim: jaesung@kirams.re.kr, Sangwook wu: s.wu@pharmcadd.com


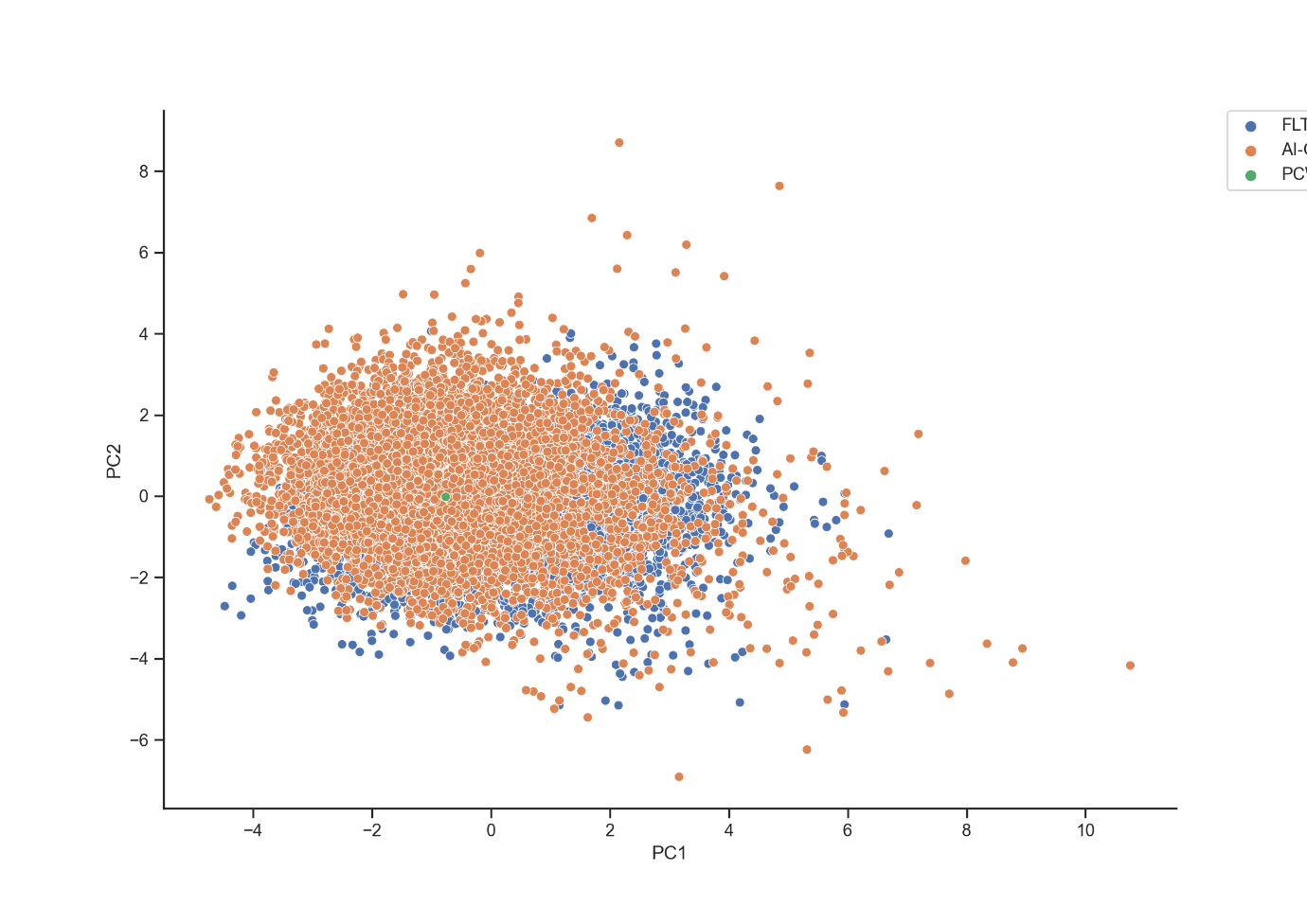


Supplementary Figure 1. Principal component analysis (PCA) applied to molecular descriptors of the FLT3 known actives, PCW-1001, and AI-generated hits. Color codes indicates, blue color dots indicate, the distribution of FLT3 Known actives (from CHEMBL), Orange color dots indicates AI-generated hits, and green dot indicates the PCW-1001. PCA analysis indicates that, AI-generated hits fall within the chemical space of known FLT3 inhibitors, and PCW-1001.

**A**


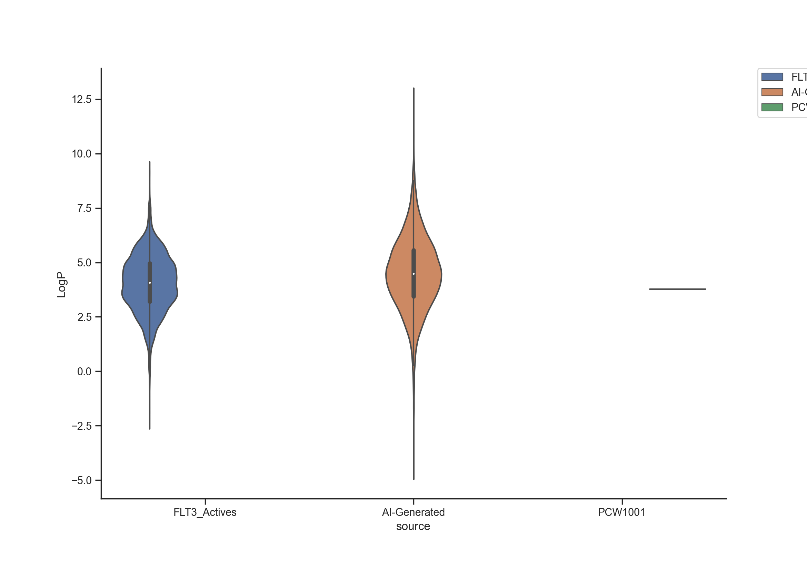


**B**


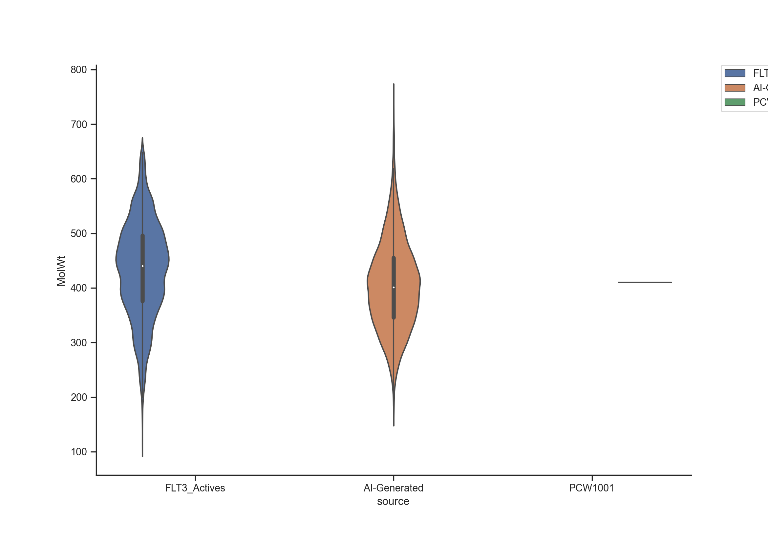


**Supplementary Figure 2.** A) violin plots indicate the distribution of LOGP of the known FLT3 actives (blue color) and AI-Generated hits (orange color) and PCW-1001 (green color); median value (white dot in the plot) of both known actives, PCW-1001 and AI generated are within the logp range of 3.5-4. 5B) violin plots indicate the distribution of Molecular weight of the known FLT3 actives (blue color) and AI-Generated hits (orange color) and PCW-1001 (green color); median value (white dot on the violin plot) of both known actives and AI generated are within the range of 400-450 daltons.


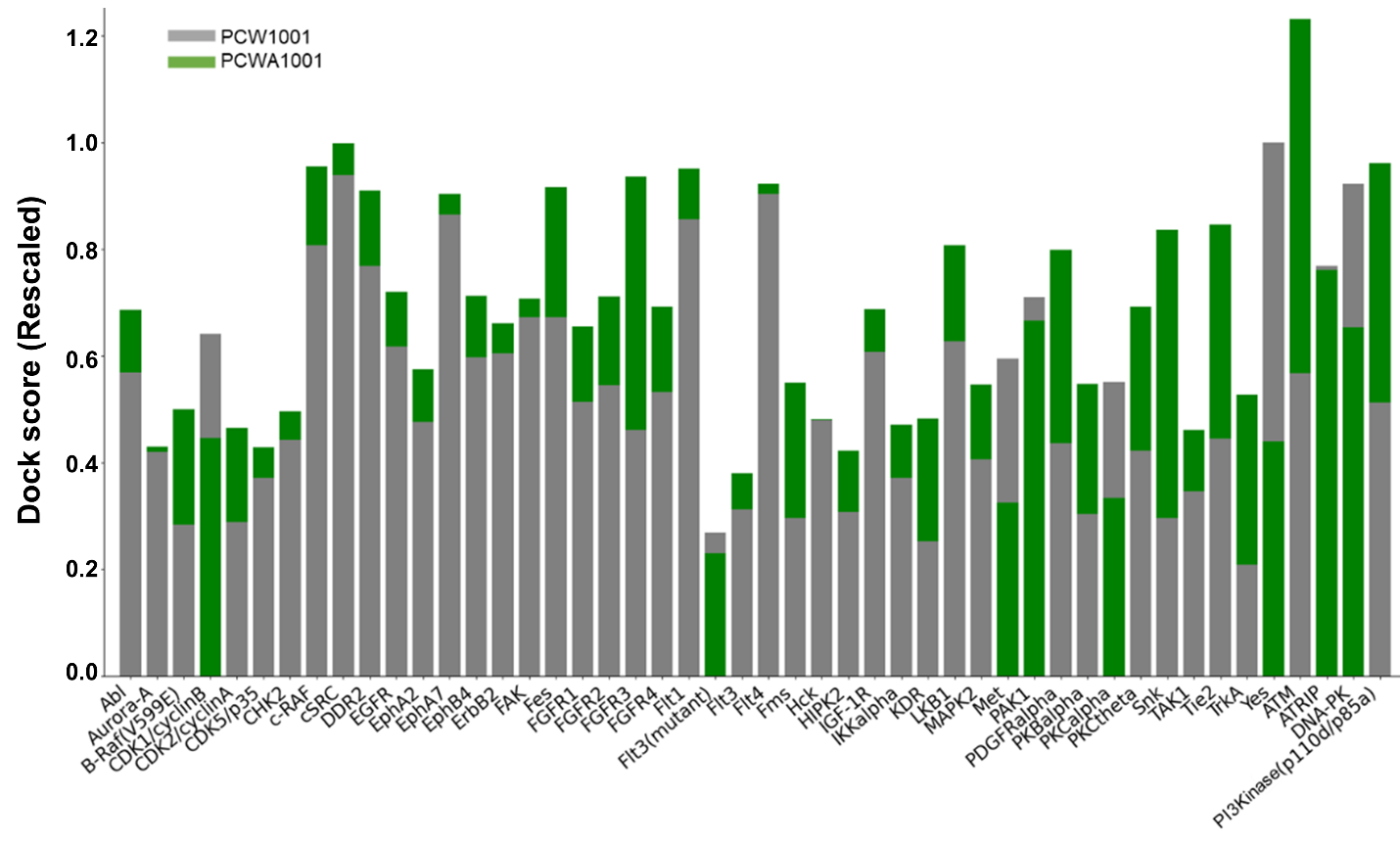
**Supplementary Figure 3.** Binding selectivity analysis of PCW-1001 and PCW-A1001 over Flt3 and Flt3(D835Y) mutant.


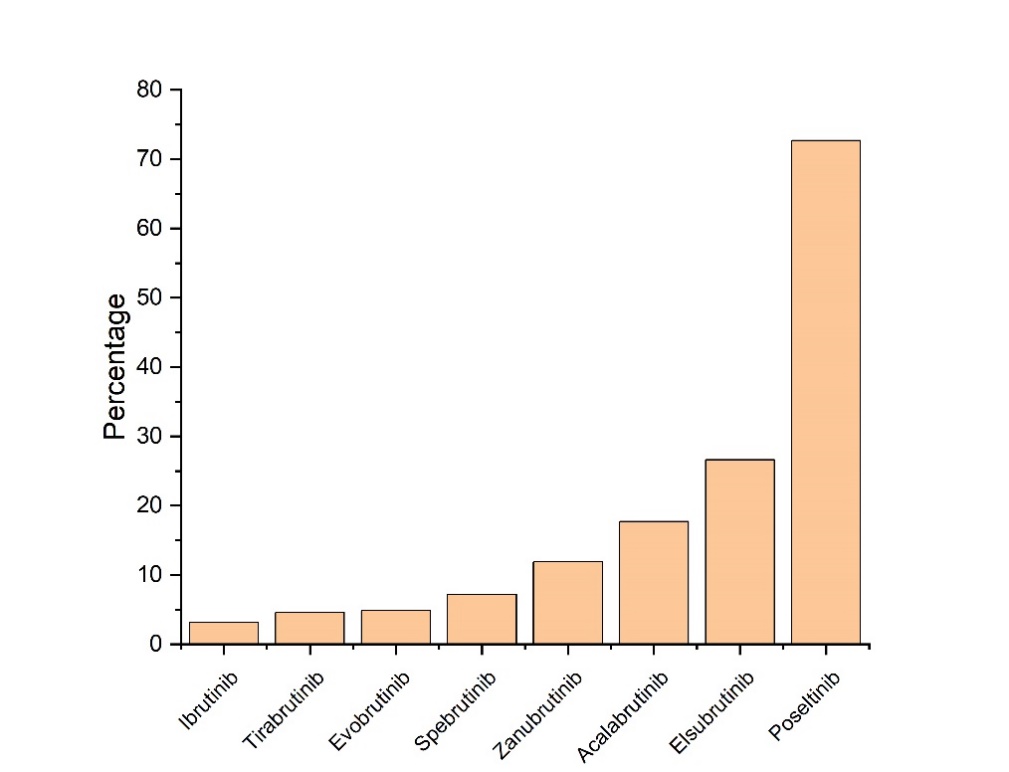


**Supplementary Figure 4.** Summary of prediction rate for selected 8 inhibitors to validate our reverse target prediction protocol.

**Supplementary Table** **1**. Validation of reverse target prediction based on the network

| **S.No.** | **Inhibitor** | **Target mentioned in Drugbank** | **Target mentioned in our network databases (PubChem gene id)** | **Results** | **Summary** |
| --- | --- | --- | --- | --- | --- |
| 1. | Ibrutinib | BTK | 1576, 1577, **`695(BTK)’**, 1565, 9263, 9262, 57118, 9201, 127933, 139728, 133690, 660, 91807, 84698, 4067, 340156, 1612, 1613, 7204, 5681, 85366, 23604, 85481, 8536, 814, 640, 5979, 57172, 828, 65975 | Shortlisted -286 proteins. Our target BTK listed as top 9^th^ . | Ranked as 3/100. |
| 2. | Acalabrutinib | BTK | Not included | Shortlisted -344 proteins.  Our target BTK is listed as 61^st^. | Ranked as 18/100. |
| 3. | Zanubrutinib | BTK, EGFR, ERBB2, ERBB4, ITK, BMX, JAK2, TEC, BLK, JAK3, PTK6, FGR, FRK, LCK, TXK | Not included | Shortlisted -329 proteins.  Our target BTK is listed as 39^th^. | Ranked as 12/100. |
| 4. | Evobrutinib | BTK^*^ | Not included | Shortlisted -430 proteins.  Our target BTK is listed as 21^st^. | Ranked as 5/100. |
| 5. | Elsubrutinib | BTK^*^ | Not included | Shortlisted -304 proteins.  Our target BTK is listed as 81^st^. | Ranked as 27/100. |
| 6. | Tirabrutinib | BTK^*^ | Not included | Shortlisted -370 proteins.  Our target BTK is listed as 17^th^. | Ranked as 5/100. |
| 7. | Spebrutinib | BTK^*^ | Not included | Shortlisted -293 proteins.  Our target BTK is listed as 21^st^. | Ranked as 7/100. |
| 8. | Poseltinib | BTK^*^ | Not included | Shortlisted -330 proteins.  Our target BTK is listed as 240^th^. | Ranked as 73/100. |

* - No target information available in Drug bank, but available in PubChem.


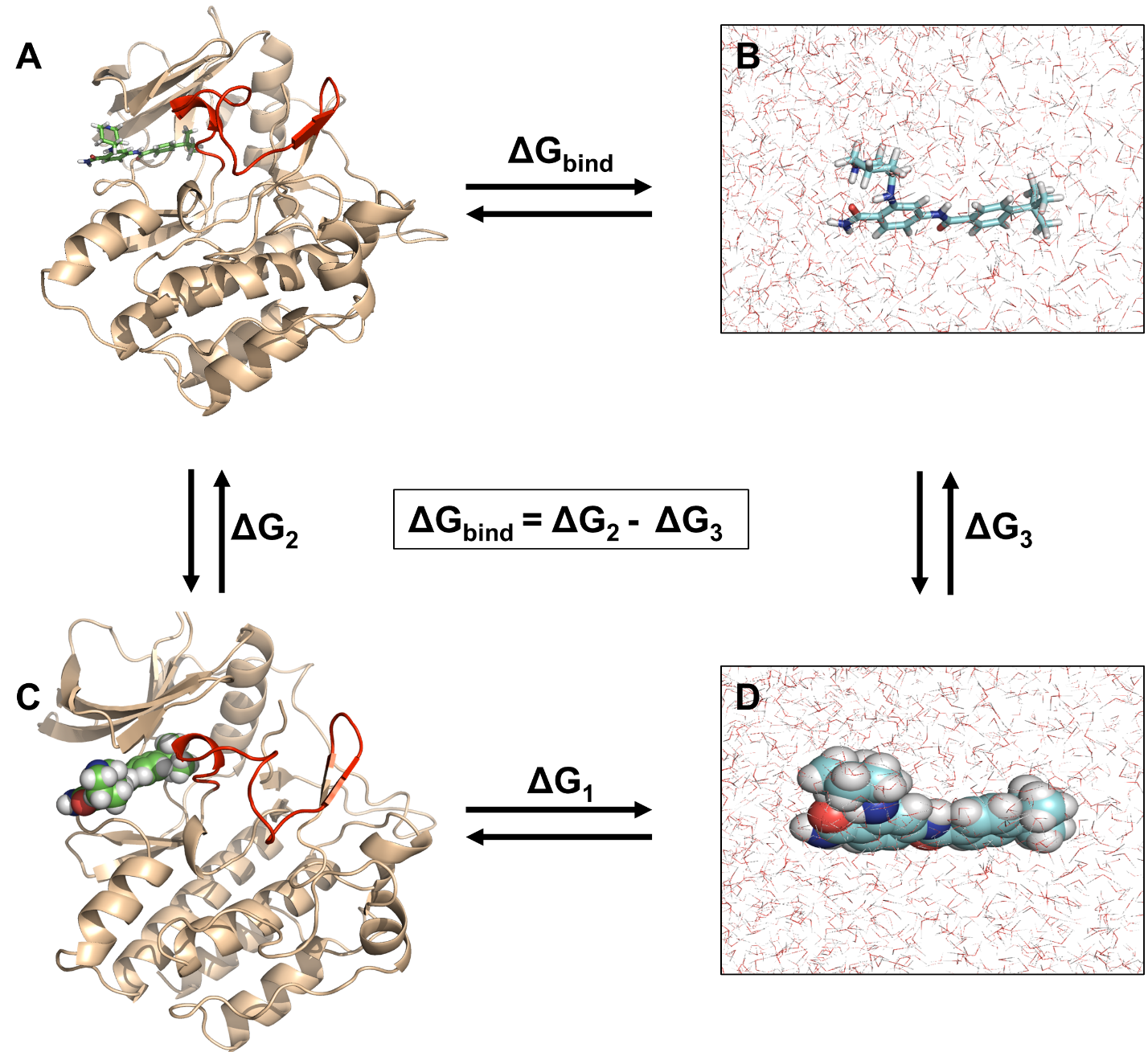


**Supplementary Figure 5. Schematic representation of an alchemical method to calculate free energy.** A) Protein and Ligand B) Ligand and Water C) Protein and dummy D) Dummy and water.

**Step by synthesis of PCW-A1001**

The synthetic routes of PCW-A1001 are summarized in Scheme 1. Methyl 2-fluoro-4-nitrobenzoate **1** and tert-butyl 3-aminopiperidine-1-carboxylate **2** were reacted in the presence of K_2_CO_3_ to obtain **3** through the nucleophilic aromatic substitution. The nitro group of compound **3** was converted to the amino functional group using hydrogenation reaction. The amine compound **4** was coupled with 4-(tert-butyl)benzoic acid in the presence of EDCI and catalytic amount of DMAP, followed by hydrolysis using LiOH·H_2_O to produce intermediate **6**. The acid functional group was converted to the amide efficiently by HBTU coupling reagent. The protecting Boc group of the secondary amine in the piperidine ring was removed by TFA to obtain desired compound **8**, PCW-A1001.

**tert-Butyl 3-((2-(methoxycarbonyl)-5-nitrophenyl)amino)piperidine-1-carboxylate(3)**

Methyl 2-fluro-4-nitrobenzoate **1** (5.0 g, 25.1 mmol) and 3-aminopiperidine-1-carboxylate **2** (5.5 g, 27.6 mmol) were dissolved in anhydrous DMF (100 mL). K_2_CO_3_ (6.9 g, 50.2 mmol) was added and the reaction mixture was stirred at 70 ℃ for 12 h. The reaction mixture was diluted with EtOAc (500 mL) and partitioned with sat’d NaHCO_3_(aq). The organic layer was collected, dried over anhydrous Na_2_SO_4_, filtered, concentrated, and purified by silica column chromatography (30 % EtOAc in hexanes) to afford compound **3** in 63 % (6.01 g) yield as a yellow solid. ^1^H-NMR (300 MHz, CDCl_3_) δ 8.15 (d, *J* = 7.5 Hz, 1H), 8.07 (d, *J* = 8.7 Hz, 1H), 7.57 (d, *J* = 2.2 Hz, 1H), 7.38 (dd, *J* = 8.8, 2.2 Hz, 1H), 3.91 (s, 3H), 3.86 – 3.81 (m, 1H), 3.62 (s, 2H), 3.38 – 3.13 (m, 2H), 2.08 (d, *J* = 12.7 Hz, 1H), 1.95 – 1.77 (m, 1H), 1.74 (d, *J* = 3.4 Hz, 2H), 1.44 (s, 9H); ^13^C-NMR (100 MHz, DMSO-*d*_6_) δ 167.6, 152.1, 150.3, 133.6, 114.5, 108.8, 106.4, 79.0, 52.8, 47.2, 47.1, 29.4, 28.2; MS (ESI) *m/z* Calcd for C_18_H_25_N_3_O_6_ (M^+^): 379.1, Found: 380.0 (M+ H^+^).

**tert-Butyl 3-((5-amino-2-(methoxycarbonyl)phenyl)amino)piperidine-1-carboxylate (4)**

tert-Butyl 3-((2-(methoxycarbonyl)-5-nitrophenyl)amino)piperidine-1-carboxylate **3** (2.0 g, 5.30 mmol) was dissolved in anhydrous EtOAc (120 mL) and Pd/C (56 mg, 0.50 mmol) was added to the reaction flask carefully. H_2_ was bubbled through the reaction mixture for 10 min, then the reaction was monitored by Thin Layer Chromatography. The reaction mixture was filtered over Celite, washing with MeOH. The filtrate was concentrated in evaporator, then dried under high vacuum to afford the compound **4** in 91 % yield (1.68 g) as a bright brown solid. ^1^H-NMR (300 MHz, DMSO-*d*_6_) δ 7.85 (s, 1H), 7.50 (d, *J* = 9.1 Hz, 1H), 5.84 (dd, *J* = 6.3, 2.4 Hz, 2H), 5.75 (s, 2H), 3.66 (s, 3H), 3.41 (s, 3H), 3.21 (s, 1H), 1.90 (s, 1H), 1.64 (s, 2H), 1.48 (s, 2H), 1.25 (s, 9H); ^13^C-NMR (100 MHz, DMSO-*d*_6_) δ 168.4, 155.1, 151.9, 133.2, 103.4, 98.7, 93.7, 78.9, 51.0, 47.6, 28.3; MS (ESI) *m/z* Calcd for C_18_H_27_N_3_O_4_ (M^+^) 349.2, Found: 350.1 (M+ H^+^).

**tert-Butyl3-((5-(4-(tert-butyl)benzamido)-2-(methoxycarbonyl)phenyl)amino)piperidine-1 carboxylate (5)**

tert-Butyl 3-((5-amino-2-(methoxycarbonyl)phenyl)amino)piperidine-1-carboxylate **4** (800 mg, 2.29 mmol), 4-(tert-butyl)benzoic acid (408 mg, 2.29 mmol) were dissolved in anhydrous DCM (23 mL) followed by the addition of EDCI (426 mg, 2.75 mmol) and DMAP (28 mg, 0.23 mmol). The reaction mixture was stirred at room temperature for 48 h. The reaction solvent was removed by evaporation and the residue was dissolved in EtOAc (120 mL), and partitioned with sat’d NaHCO_3_(aq). The organic layer was collected, dried over anhydrous Na_2_SO_4_, filtered, concentrated, and purified by silica column chromatography (20 % EtOAc in hexanes) to afford compound **5** in 90 % (1.06 g) yield as a light-yellow solid. ^1^H-NMR (300 MHz, DMSO-*d*_6_) δ 10.23 (s, 1H), 7.88 (d, *J* = 8.4 Hz, 3H), 7.78 (d, *J* = 8.8 Hz, 1H), 7.56 (d, *J* = 8.4 Hz, 2H), 7.04 (s, 1H), 3.76 (s, 3H), 3.60 – 3.47 (m, 3H), 3.42 (s, 1H), 1.95 (s, 1H), 1.68 (s, 2H), 1.53 (s, 2H), 1.33 (s, 9H), 1.30 – 1.15 (m, 9H); ^13^C-NMR (100 MHz, DMSO-*d*_6_) δ 168.2, 166.3, 155.1, 150.9, 145.6, 132.4, 129.6, 128.0, 125.6, 107.6, 105.3, 102.1, 51.7, 47.4, 47.2, 35.1, 31.3, 31.3, 29.6, 28.1; MS (ESI) *m/z* Calcd for C_29_H_39_N_3_O_5_ (M^+^) 509.2, Found: 510.1 (M+ H^+^).

**2-((1-(tert-Butoxycarbonyl)piperidin-3-yl)amino)-4-(4-(tert-butyl)benzamido)benzoic acid (6)**

tert-Butyl 3-((5-(4-(tert-butyl)benzamido)-2-(methoxycarbonyl)phenyl)amino)piperidine-1 carboxylate **5** (100 mg, 0.20 mmol) was dissolved in THF/MeOH/H_2_O (5 mL, ratio=3/1/1) and LiOH·H_2_O (24 mg, 0.60 mmol) was added to the reaction vessel. The reaction mixture was stirred at room temperature for 12 h. The reaction mixture was diluted with further water, acidified to pH 3 with 1N HCl and then extracted into EtOAc (x5). The organic layers were combined, dried over Na_2_SO_4_, filtered and concentrated to afford compound **6** in 88 % (86 mg) yield as a pale-white solid. ^1^H-NMR (300 MHz, DMSO-*d*_6_) δ 10.03 (s, 1H), 8.96 (s, 1H), 7.92 – 7.85 (m, 2H), 7.76 (d, *J* = 8.5 Hz, 1H), 7.57 – 7.49 (m, 2H), 7.29 (s, 1H), 6.87 (s, 1H), 3.78 (d, *J* = 12.4 Hz, 2H), 3.62 – 3.48 (m, 2H), 3.10 (s, 2H), 1.99 (s, 1H), 1.71 (s, 1H), 1.51 (d, *J* = 17.4 Hz, 1H), 1.33 (s, 9H), 1.29 (s, 9H); ^13^C-NMR (100 MHz, DMSO-*d*_6_) δ 171.3, 165.9, 154.8, 154.3, 150.2, 143.2, 132.9, 132.8, 127.9, 125.5, 106.7, 102.1, 78.9, 48.2, 47.9, 35.1, 31.4, 30.7, 28.3, 23.3; MS (ESI) *m/z* Calcd for C_28_H_37_N_3_O_5_ (M^+^)495.1 , Found: 496.2 (M+ H^+^).

**tert-Butyl3-((5-(4-(tert-butyl)benzamido)-2-carbamoylphenyl)amino)piperidine-1-carboxylate (7)**

2-((1-(tert-Butoxycarbonyl)piperidin-3-yl)amino)-4-(4-(tert-butyl)benzamido)benzoic acid **6** (80 mg, 0.16 mmol), NH_4_Cl (25 mg, 0.48 mmol) were added to the flame-dried reaction flask containing anhydrous DMF (2 mL). HBTU (91 mg, 0.24 mmol) and DIPEA (83 µL, 0.48 mmol) were added to the reaction flask. The reaction mixture was stirred at room temperature for 12 h. The reaction mixture was diluted with EtOAc (100 mL), and partitioned with sat’d NaHCO_3_(aq). The organic layer was collected, dried over anhydrous Na_2_SO_4_, filtered, concentrated, and purified by silica column chromatography (50 % EtOAc in hexanes) to afford compound **7** in 78 % (62 mg) yield as a brown solid. ^1^H-NMR (300 MHz, DMSO-*d*_6_) δ 10.10 (s, 1H), 8.59 (s, 1H), 7.88 (d, *J* = 8.3 Hz, 2H), 7.70 (s, 1H), 7.61 (d, *J* = 8.7 Hz, 1H), 7.55 (d, *J* = 8.4 Hz, 2H), 7.31 (d, *J* = 26.9 Hz, 1H), 6.97 (s, 2H), 3.65 (d, *J* = 12.5 Hz, 1H), 3.39 (s, 2H), 3.26 (s, 1H), 1.97 (d, *J* = 11.5 Hz, 1H), 1.68 (s, 2H), 1.51 (d, *J* = 15.6 Hz, 2H), 1.33 (s, 9H), 1.27 (s, 9H); ); ^13^C-NMR (100 MHz, DMSO-*d*_6_) δ 171.6, 166.0, 155.0, 150.0, 143.7, 132.6, 130.3, 127.9, 125.6, 109.7, 106.7, 102.5, 78.9, 47.8, 47.6, 35.1, 31.4, 30.2, 28.2; MS (ESI) *m/z* Calcd for C_28_H_38_N_4_O_4_ (M^+^) 494.3 , Found: 495.3 (M+ H^+^).

**4-(tert-Butyl)-N-(4-carbamoyl-3-(piperidin-3-ylamino)phenyl)benzamide (8, PCW-A1001)**

tert-Butyl3-((5-(4-(tert-butyl)benzamido)-2-carbamoylphenyl)amino)piperidine-1-carboxylate (50 mg, 0.10 mmol) was dissolved in 20 % TFA/DCM (2 mL). After stirring at room temperature for 3 h, TLC indicated the reaction was complete. All solvents were removed in vacuo and residual TFA was removed by repeated azeotroping with CHCl_3_. The residue was dried under high vacuum to deliver the title compound **8** in 63 % (25 mg) yield as a white solid. ^1^H-NMR (300 MHz, DMSO-*d_6_*) δ 10.23 (s, 1H), 9.12 (s, 2H), 8.51 (d, *J* = 7.3 Hz, 1H), 7.92 (d, *J* = 8.2 Hz, 2H), 7.81 (s, 1H), 7.65 (d, *J* = 8.9 Hz, 1H), 7.55 (d, *J* = 8.1 Hz, 2H), 7.40 (d, *J* = 3.5 Hz, 1H), 7.12 (s, 1H), 7.01 (d, *J* = 8.6 Hz, 1H), 3.68 (s, 1H), 3.51 – 3.41 (m, 1H), 3.20 (d, *J* = 12.4 Hz, 1H), 2.86 (dt, *J* = 22.0, 11.6 Hz, 2H), 2.10 (d, *J* = 12.1 Hz, 1H), 1.82 (d, *J* = 31.7 Hz, 2H), 1.51 (d, *J* = 12.3 Hz, 1H), 1.32 (s, 9H); ^13^C-NMR (100 MHz, DMSO-d*_6_*) δ 171.66, 166.21, 155.10, 149.55, 143.76, 132.54, 130.46, 128.01, 125.68, 110.03, 107.51, 102.56, 46.78, 46.22, 43.42, 35.17, 31.38, 29.23, 21.23; MS (ESI) *m/z* Calcd for C_23_H_30_N_4_O_2_ (M^+^) 394.2, Found: 395.1 (M+ H^+^); HRMS (EI^+^) m/z Calcd for C_23_H_30_N_4_O_2_ (M^+^) 394.2369, Found: 394.2350.

**
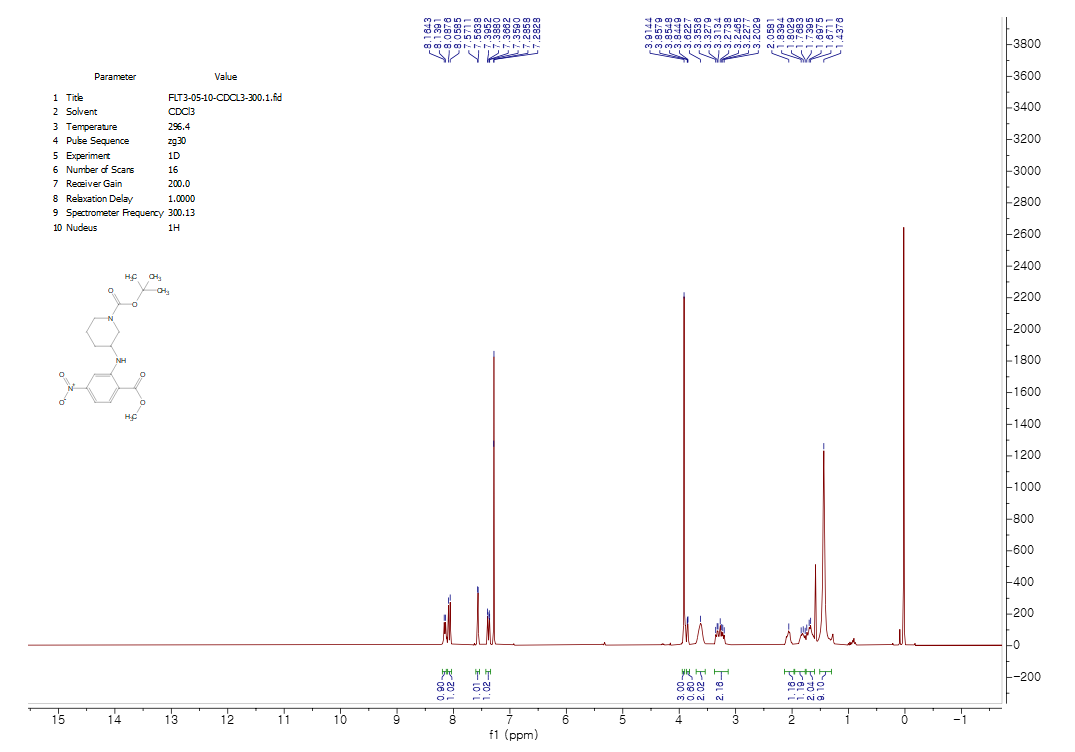
Supplementary Figure 6.** 1H-NMR (300 MHz, CDCl3) spectrum of compound 3: tert-butyl 3-((2-(methoxycarbonyl) -5-nitrophenyl)amino)piperidine-1-carboxylate.

**
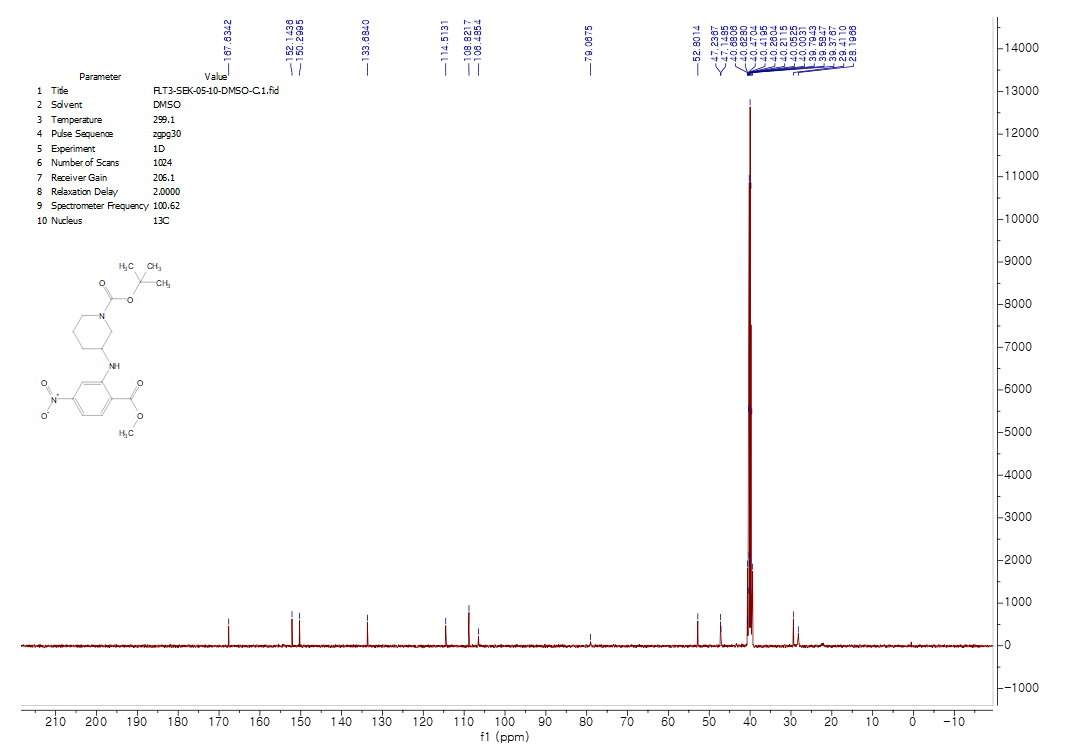
 Supplementary Figure 7**. ^13^C-NMR (100 MHz, DMSO-*d*_6_) spectrum of compound **3**: tert-butyl 3-((2-(methoxycarbonyl)-5-nitrophenyl)amino)piperidine-1-carboxylate.

**
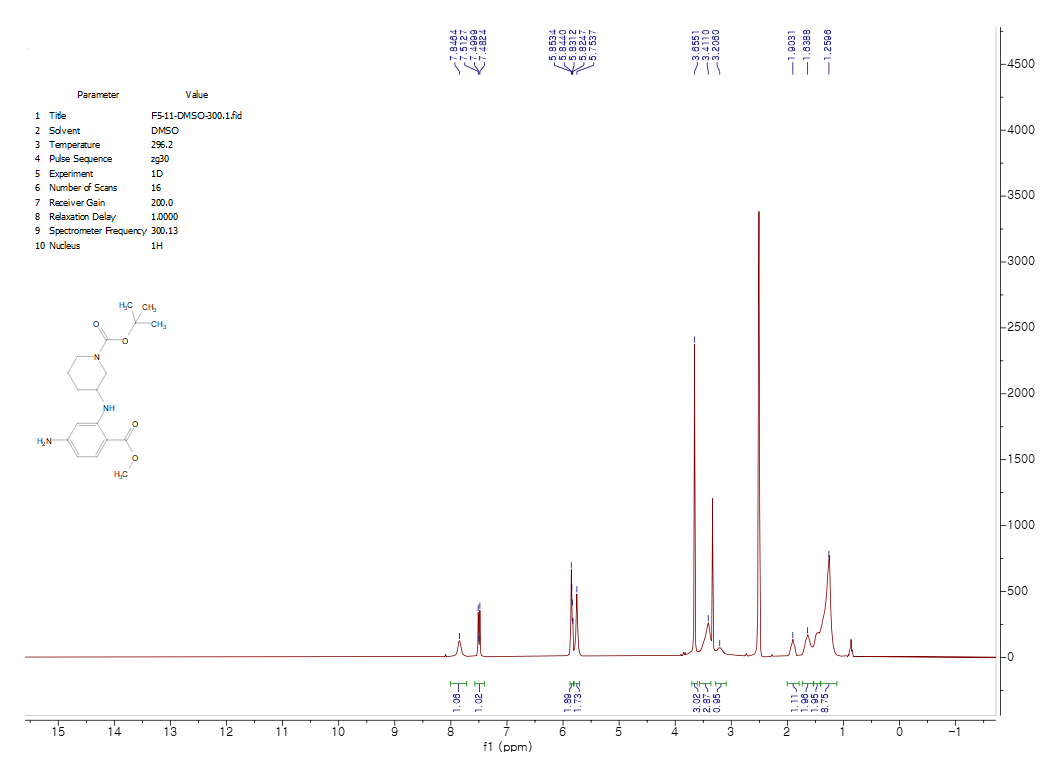
 Supplementary Figure 8**. 1H-NMR (300 MHz, DMSO-d6) spectrum of compound 4: tert-butyl 3-((5-amino-2-(methoxycarbonyl)phenyl)amino)piperidine-1-carboxylate.


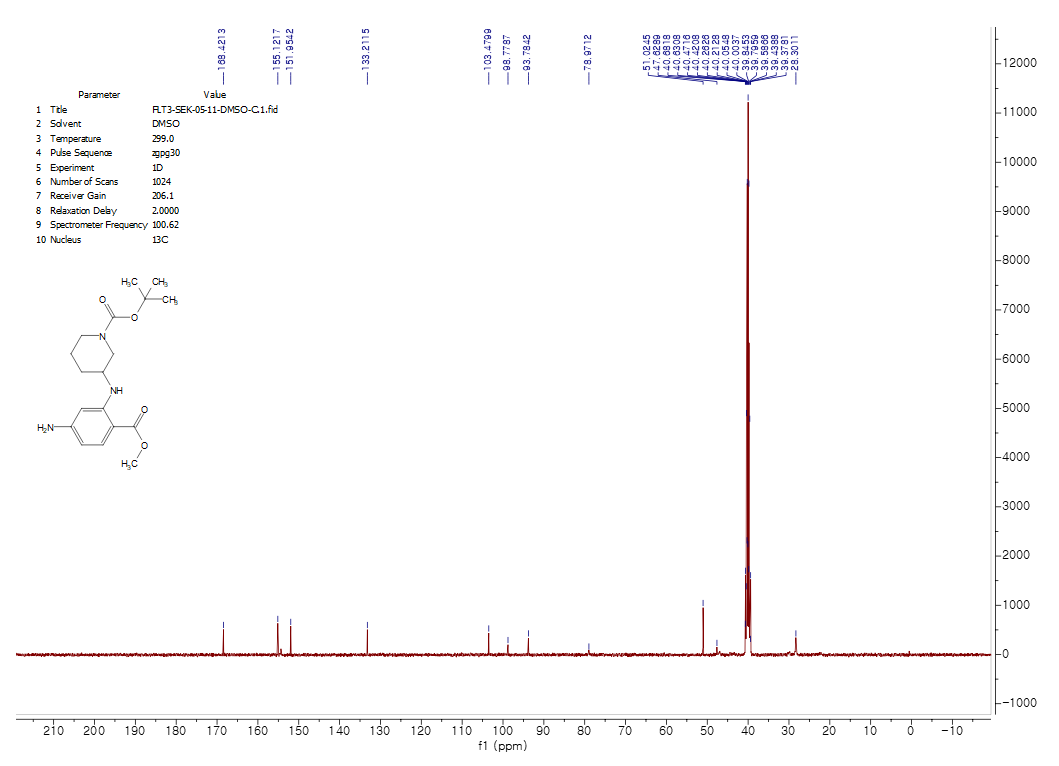
 **Supplementary Figure 9**. ^13^C-NMR (100 MHz, DMSO-*d*_6_) spectrum of compound **4**: tert-butyl 3-((5-amino-2-(methoxycarbonyl)phenyl)amino)piperidine-1-carboxylate.

**
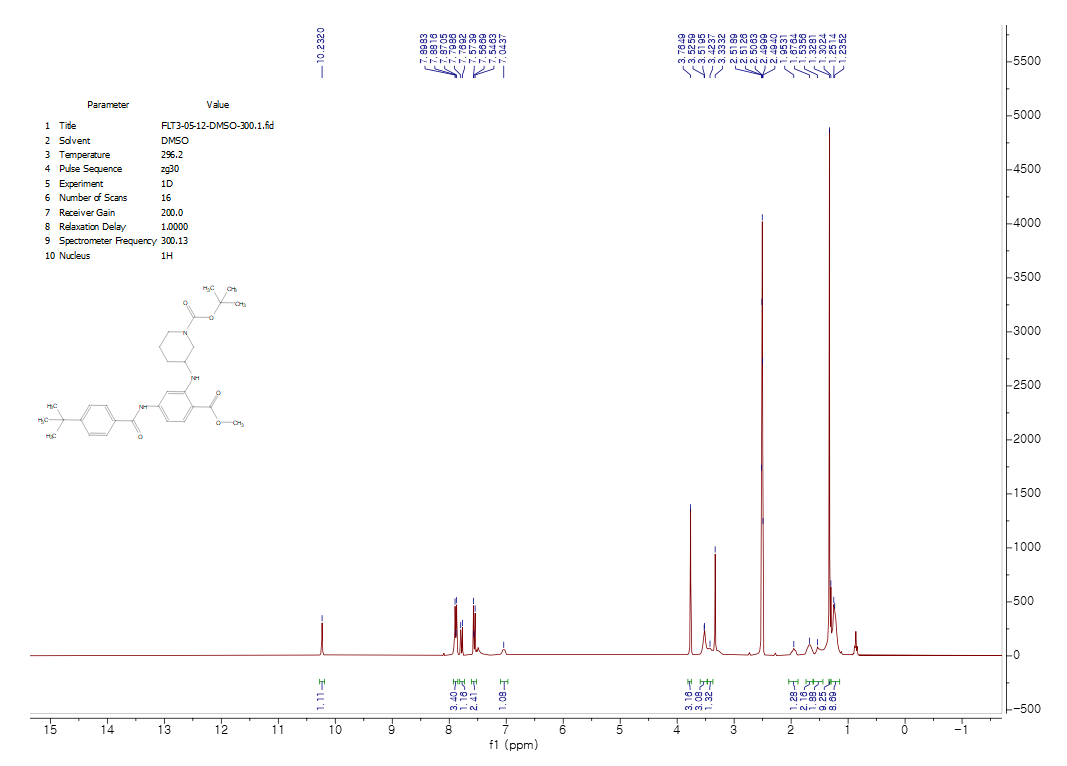
 Supplementary Figure 10**. 1H-NMR (300 MHz, DMSO-d6) spectrum of compound 5: tert-butyl 3-((5-(4-(tert-butyl)benzamido)-2-(methoxycarbonyl)phenyl)amino)piperidine-1-carboxylate.

**
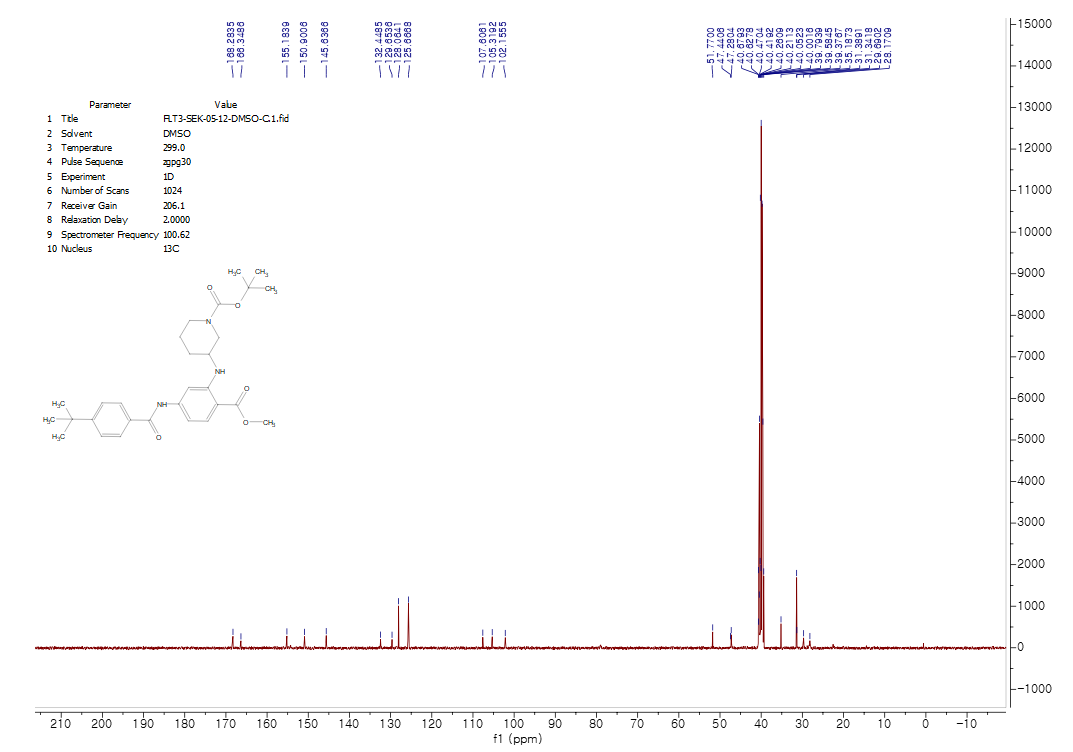
 Supplementary Figure 11.** ^13^C-NMR (100 MHz, DMSO-*d*_6_) spectrum of compound **5**: tert-butyl 3-((5-(4-(tert-butyl)benzamido)-2-(methoxycarbonyl)phenyl)amino)piperidine-1-carboxylate.

**
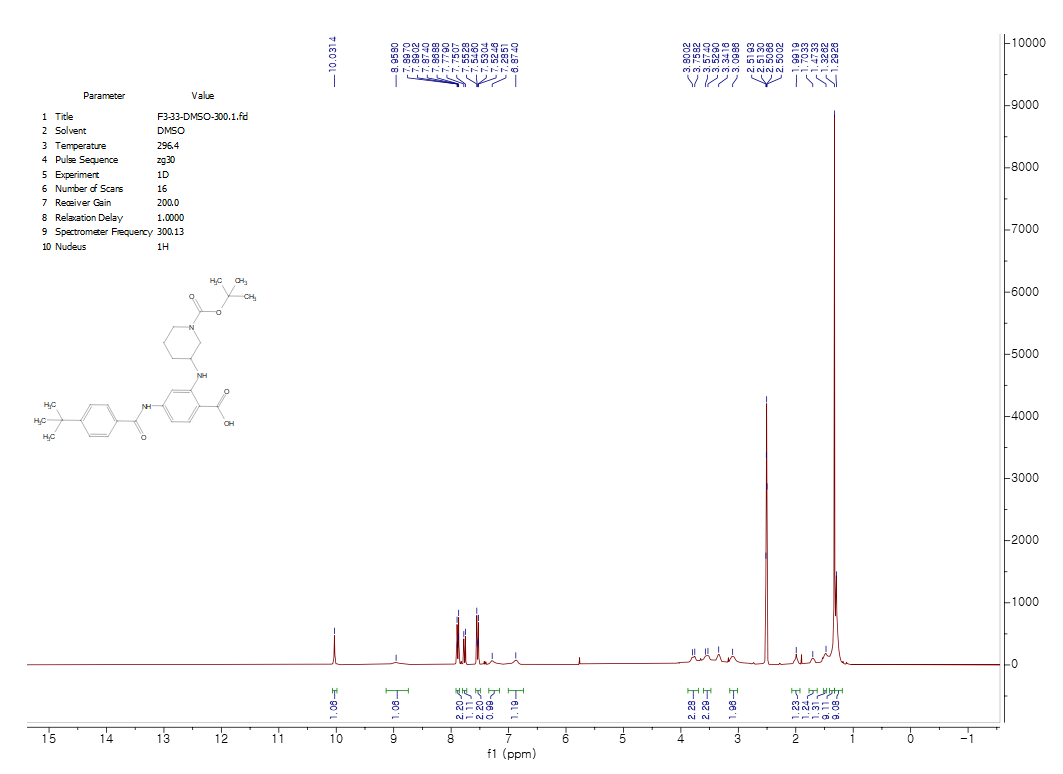
 Supplementary Figure 12**. 1H-NMR (300 MHz, DMSO-d6) spectrum of compound 6: 2-((1-(tert-butoxycarbonyl)piperidin-3-yl)amino)-4-(4-(tert-butyl)benzamido)benzoic acid.


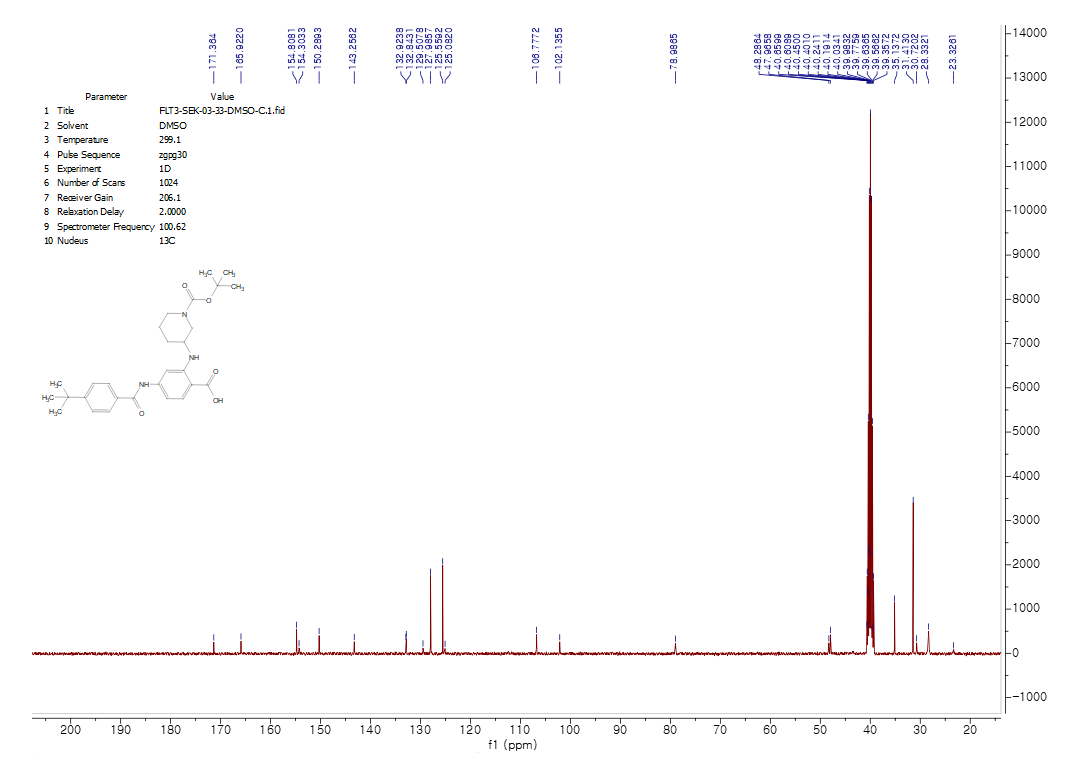
 **Supplementary Figure 13**. ^13^C-NMR (100 MHz, DMSO-*d*_6_) spectrum of compound **6**: 2-((1-(tert-butoxycarbonyl)piperidin-3-yl)amino)-4-(4-(tert-butyl)benzamido)benzoic acid.

**
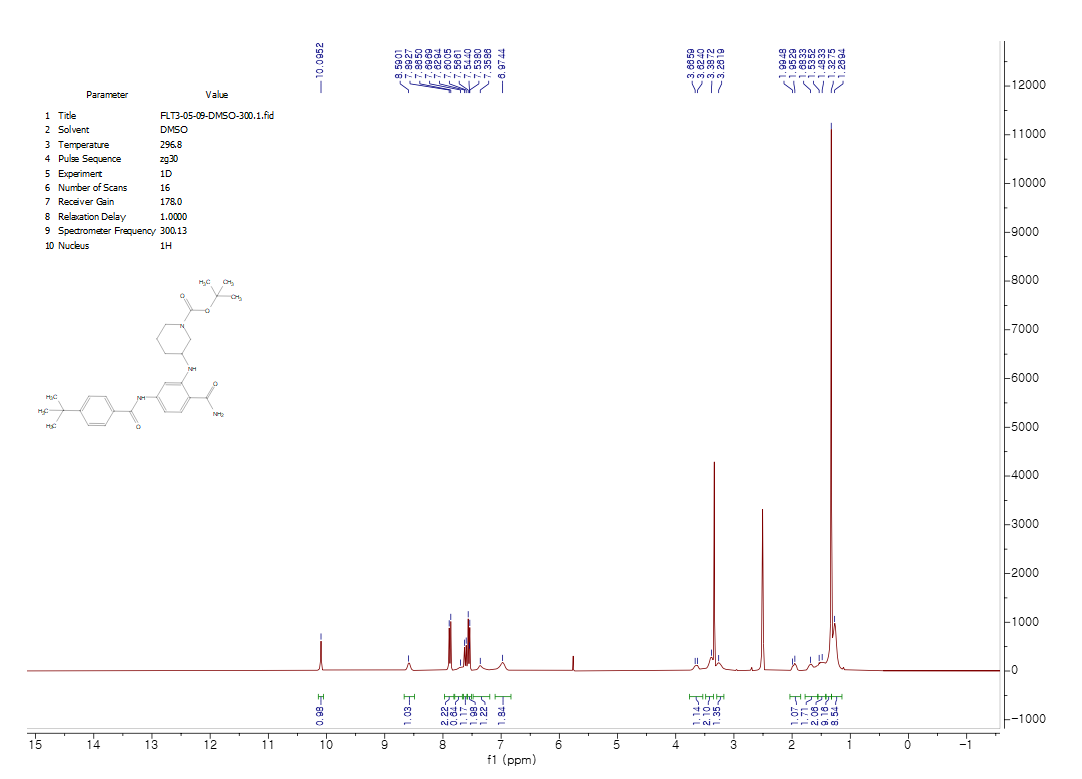
 Supplementary Figure 14**. 1H-NMR (300 MHz, DMSO-d6) spectrum of compound 7: tert-butyl 3-((5-(4-(tert-butyl)benzamido)-2-carbamoylphenyl)amino)piperidine-1-carboxylate.


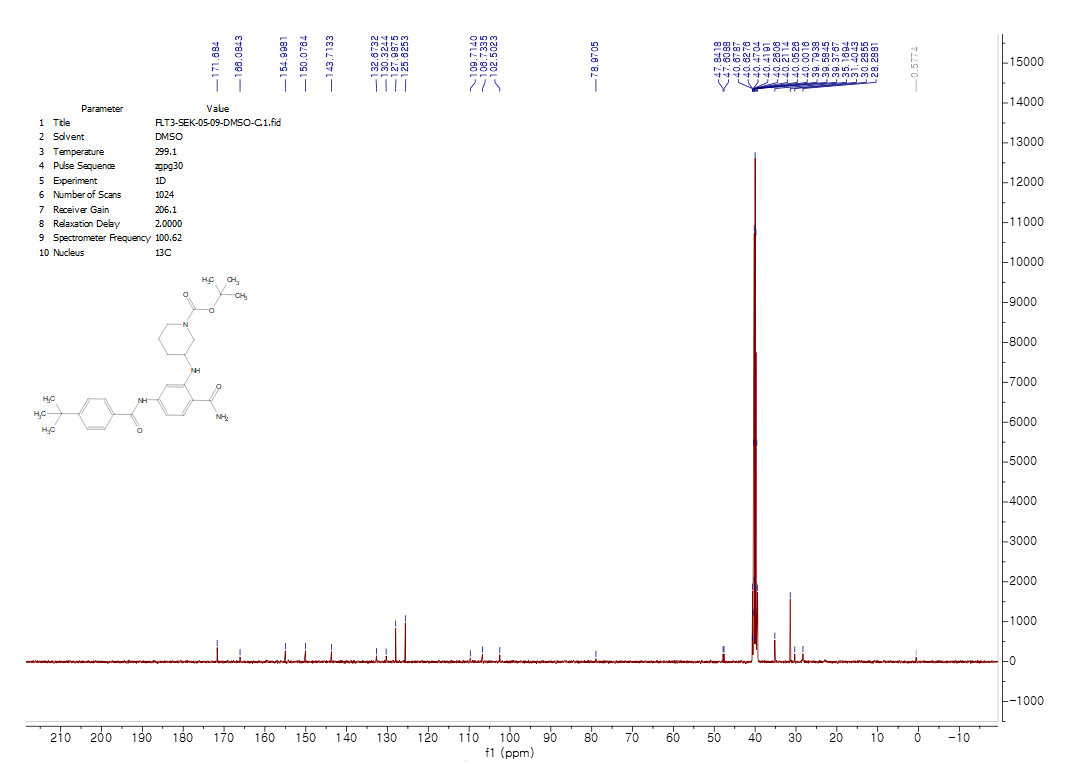
 **Supplementary Figure 15**. ^13^C-NMR (100 MHz, DMSO-*d*_6_) spectrum of compound **7**: tert-butyl 3-((5-(4-(tert-butyl)benzamido)-2-carbamoylphenyl)amino)piperidine-1-carboxylate.

**^
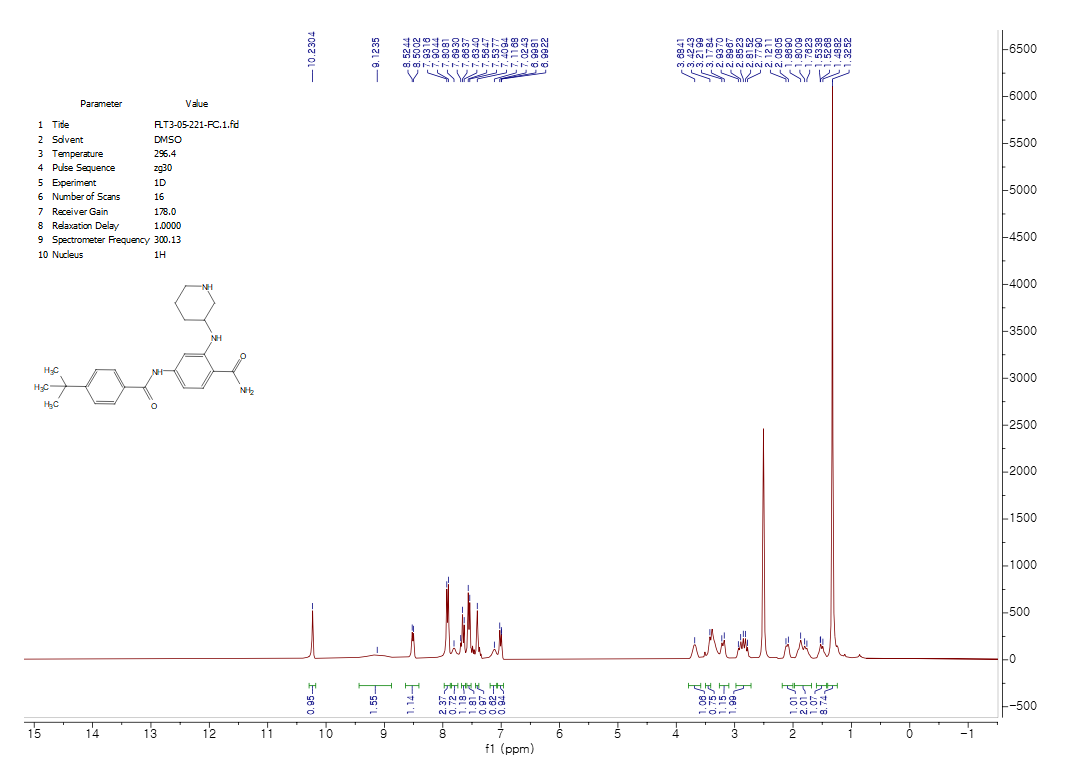
^ Supplementary Figure 16**. 1H-NMR (300 MHz, DMSO-d6) spectrum of compound 8 (PCW-A1001): 4-(tert-butyl)-N-(4-carbamoyl-3-(piperidin-3-ylamino)phenyl)benzamide.

**
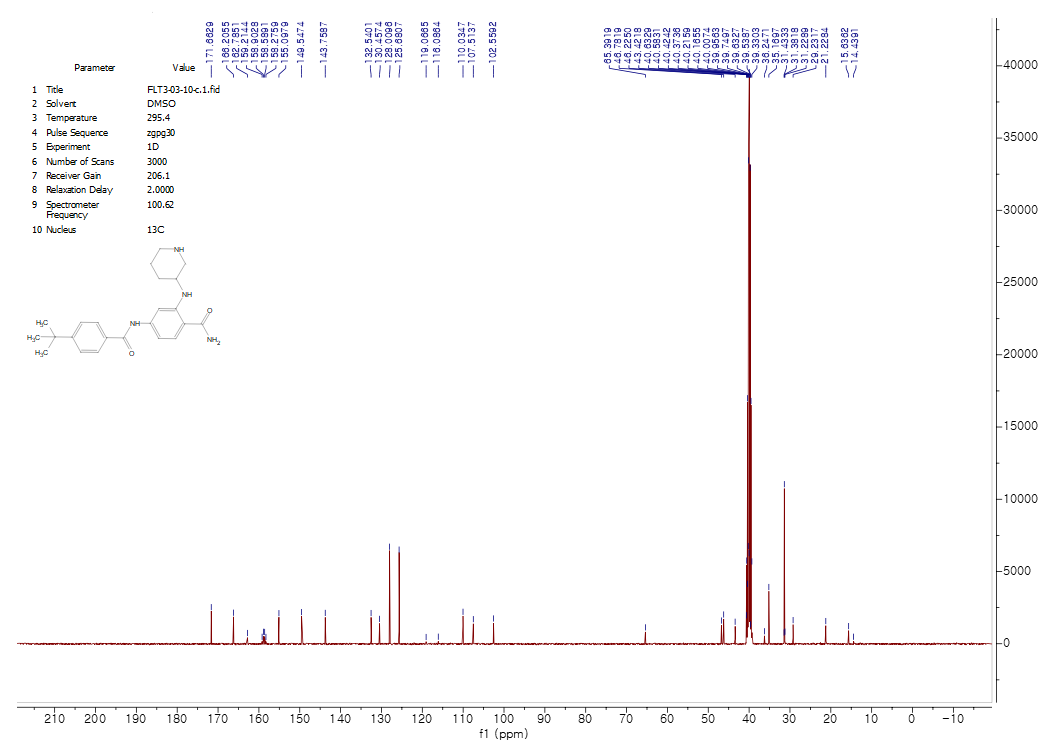
 Supplementary Figure 17**. 13C-NMR (100 MHz, DMSO-d6) spectrum of compound 8 (PCW-A1001): 4-(tert-butyl)-N-(4-carbamoyl-3-(piperidin-3-ylamino)phenyl)benzamide.


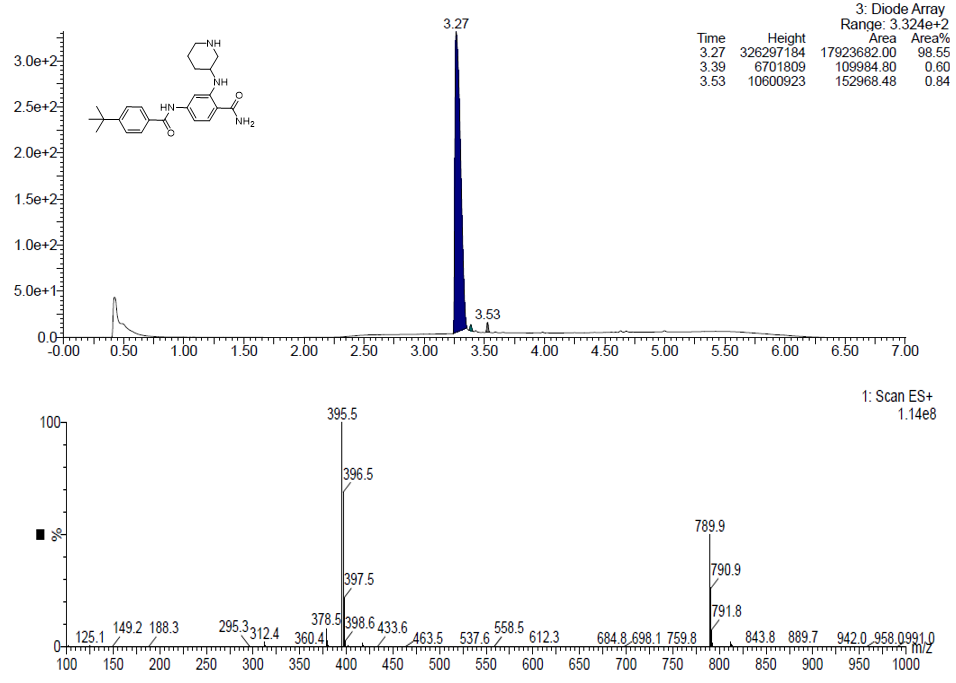
 **Supplementary Figure 18**. ESI-LC-MS spectrum of compound 8 (PCW-A1001): 4-(tert-butyl)-N-(4-carbamoyl-3-(piperidin-3-ylamino)phenyl)benzamide.

**
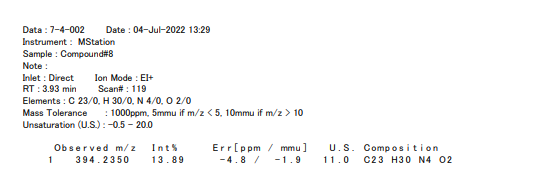
 Supplementary Figure 19**. HRMS of compound **8** (PCW-A1001): 4-(tert-butyl)-N-(4-carbamoyl-3-(piperidin-3-ylamino)phenyl)benzamide
